# Emerging diseases of *Cannabis sativa* and sustainable management

**DOI:** 10.1002/ps.6307

**Published:** 2021-02-27

**Authors:** Zamir K Punja

**Affiliations:** ^1^ Department of Biological Sciences Simon Fraser University Burnaby BC Canada

**Keywords:** biocontrol, disease management, fungal pathogens, hemp, marijuana, plant pathology

## Abstract

Cultivation of cannabis plants (*Cannabis sativa* L., marijuana) has taken place worldwide for centuries. In Canada, legalization of cannabis in October 2018 for the medicinal and recreational markets has spurned interest in large‐scale growing. This increased production has seen a rise in the incidence and severity of plant pathogens, causing a range of previously unreported diseases. The objective of this review is to highlight the important diseases currently affecting the cannabis and hemp industries in North America and to discuss various mitigation strategies. Progress in molecular diagnostics for pathogen identification and determining inoculum sources and methods of pathogen spread have provided useful insights. Sustainable disease management approaches include establishing clean planting stock, modifying environmental conditions to reduce pathogen development, implementing sanitation measures, and applying fungal and bacterial biological control agents. Fungicides are not currently registered for use and hence there are no published data on their efficacy. The greatest challenge remains in reducing microbial loads (colony‐forming units) on harvested inflorescences (buds). Contaminating microbes may be introduced during the cultivation and postharvest phases, or constitute resident endophytes. Failure to achieve a minimum threshold of microbes deemed to be safe for utilization of cannabis products can arise from conventional and organic cultivation methods, or following applications of beneficial biocontrol agents. The current regulatory process for approval of cannabis products presents a challenge to producers utilizing biological control agents for disease management. © 2021 The Author. *Pest Management Science* published by John Wiley & Sons Ltd on behalf of Society of Chemical Industry.

## Graphical abstract

Pathogen inoculum originating from fields cropped to blueberry, tomato, barley, garlic and cabbage can spread to adjacent crops of cannabis and hemp, and cause infection and subsequent disease development.

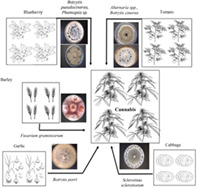

## 1 INTRODUCTION

The family Cannabaceae contains three important plant species that are cultivated world‐wide: (i) cannabis (*Cannabis sativa*, marijuana), grown for its medicinal and psychotropic properties, which are attributed to cannabinoid and terpene compounds produced in the female inflorescences,^1^, ^2^, ^3^, ^4^ (ii) hemp (*C. sativa*), grown as a source of fibre and oilseed present in stems and flowers,^4^ respectively, and more recently for its cannabinoids, and (iii) hops (*Humulus lupulus*), cultivated for the female cones that produce aromatic oils and alpha acids used in the brewing industry.^5^ In Canada, producers of both cannabis and hemp must follow guidelines established by Health Canada to ensure the final products meet specific requirements for safety and quality.^6^ For example, hemp plants must have a delta‐9‐tetrahydrocannabinol (THC) content that does not exceed 0.3% (by dry weight); THC levels in cannabis plants may range from <10% to >20% in the dried inflorescences. A wide variety of other cannabinoids, terpene and phenolic compounds are also present in hemp and cannabis.^1^, ^2^, ^3^, ^4^ Dried cannabis products must have minimal contamination of the inflorescences (buds) by fungi, yeasts and bacteria, as well as by specific coliform bacteria, chemical pesticides and mycotoxins.^7^, ^8^, ^9^ Products failing to meet the minimum threshold requirement for these contaminants cannot be sold. Limits imposed for culturable colony‐forming units (cfu/g) of yeast and mold vary depending on specific countries, and can range from <1000 to >10 000 cfu/g.^6^, ^10^

The greatest challenge facing cannabis and hemp producers is the management of insect pests and pathogens that attack the roots, leaves and inflorescences. With the rapidly expanding cultivation of these crops in Canada, accompanied by a rapid expansion of hemp cultivation in the USA following implementation of the Farm Bill of 2018, producers and researchers face challenges in the identification and management of newly appearing (previously unreported) diseases as well as insect pests. Descriptions of the diseases affecting hemp and cannabis over the period of 1990–2000 are available.^8^, ^11^, ^12^, ^13^, ^14^ Using symptomology, morphological criteria and molecular approaches for pathogen identification, these descriptions of cannabis and hemp diseases are the most comprehensive currently available. In the present review, further characterization of newly emerging pathogens of cannabis and hemp reported over the period 2017–2020 is summarized (see Table S1) and approaches to disease management are discussed (Table 1).

**Table 1:** The most important pathogens currently affecting cannabis production indoors and management practices

Common name of disease	Pathogen(s)	Management options
Damping‐off	*Botrytis cinerea* *Fusarium oxysporum* *Fusarium proliferatum* *Fusarium solani*	Reduce ambient relative humidity, improve air circulation Apply biological control agents at rooting Removed diseased cuttings
Fusarium root and crown rot	*Fusarium oxysporum* *Fusarium proliferatum* *Fusarium solani*	Stock (mother) plants to be tested to ensure they are pathogen‐free Apply biological control agents at the vegetative stage of growth Avoid injury to roots and overwatering
Pythium root and crown rot	*Pythium myriotylum* *Pythium dissotocum* *Pythium aphanidermatum*	Avoid excessive watering Avoid injury to roots Apply biological control agents at rooting and vegetative stages of growth
Powdery mildew	*Golovinomyces* spp.	Vegetative cuttings should be disease‐free Irradiate leaves for 3–4 s with UV‐C light daily Apply weekly treatments of potassium bicarbonate Grow strains that are tolerant to infection Vaporize sulfur at night Remove and destroy diseased leaves
Bud rots	*Botrytis cinerea* *Fusarium* spp.	Reduce ambient humidity and moisture Avoid growing strains with large dense inflorescences that retain moisture Prune out diseased buds and destroy them
Post‐harvest molds	*Botrytis cinerea* *Penicillium* species	Maintain drying room conditions at optimal humidity and temperature Avoid damage to buds during harvesting and trimming Irradiate dried buds with gamma or electrobeam radiation
Dudding	*Hop latent viroid*	Stock plants to be tested to confirm they are pathogen‐free Remove and destroy infected plants

Canada and Uruguay are presently the only two countries that have fully legalized the cultivation, sale, possession and consumption of cannabis and its by‐products nationwide. The recent regulatory approvals granted in 2018 in Canada (for recreational cannabis) and the USA (by passage of the Farm Bill of 2018 allowing domestic hemp production) has meant there has been insufficient lead time to assess and approve chemical pesticides for pest and disease management. The Canadian Pest Management Regulatory Agency (PMRA) and the US Environmental Protection Agency (EPA) have approved only two chemical pesticides that producers may legally apply to their crops at the present time. In Canada, vaporized sulfur is permitted indoors, while in the USA, potassium salts of fatty acids are registered as a pesticide. Consequently, efficacy data on any other fungicides are lacking. Fortunately, there are many microbial biological agents (biopesticides), as well as reduced risk products (such as potassium bicarbonate and hydrogen peroxide) which have been approved and are registered for use on hemp and cannabis. Even then, data on their comparative efficacy and field evaluations are currently lacking in the peer‐reviewed literature. In this review, the approaches to sustainable disease management using biologicals and reduced risk products will be emphasized.

## 2 CANNABIS CULTIVATION

Historically, cannabis cultivation began in outdoor growing in areas where climatic conditions allowed plants to grow for at least 120 days to complete flowering.^4^ This was followed by indoor cultivation in growing facilities with controlled environmental conditions and supplemental lighting to optimize plant growth. Most indoor cultivation currently utilizes hydroponic soil‐free culture, e.g. rockwool or cocofibre, although soil culture is common, especially for organic production. A combination of indoor environments, which includes expansive greenhouse production, and outdoor (field) environments is used to cultivate cannabis in Canada. Each production environment faces challenges from plant pathogens, with indoor and greenhouse systems sharing more diseases in common compared to field‐grown cannabis. The nature of these diseases is described in more detail in this review.

Production statistics for cannabis in Canada in 2020 indicate there are 308 license holders for cultivation conducted over 1.9 million square meters of indoor and greenhouse space, and 46 license holders for outdoor production on 544 ha of land. For hemp, production currently occurs by 700 license holders over 31 500 ha in Canada. In the USA, 16 800 growers were issued licenses in 2019 in 34 states for hemp cultivation on over 202 000 ha. Since cannabis is not approved at the federal level, production statistics are unavailable despite more than 36 US states having legalized recreational use of cannabis. Worldwide, the USA ranks as the top cannabis producer, followed by Morocco, Afghanistan, Mexico, Columbia, Paraguay, Jamaica and Canada. The small number of countries in which cannabis can be legally cultivated has subsequently limited the availability of peer‐reviewed research on aspects of pest and disease management. This review includes recently published reports over the period 2017–2020.

**Figure 1:**
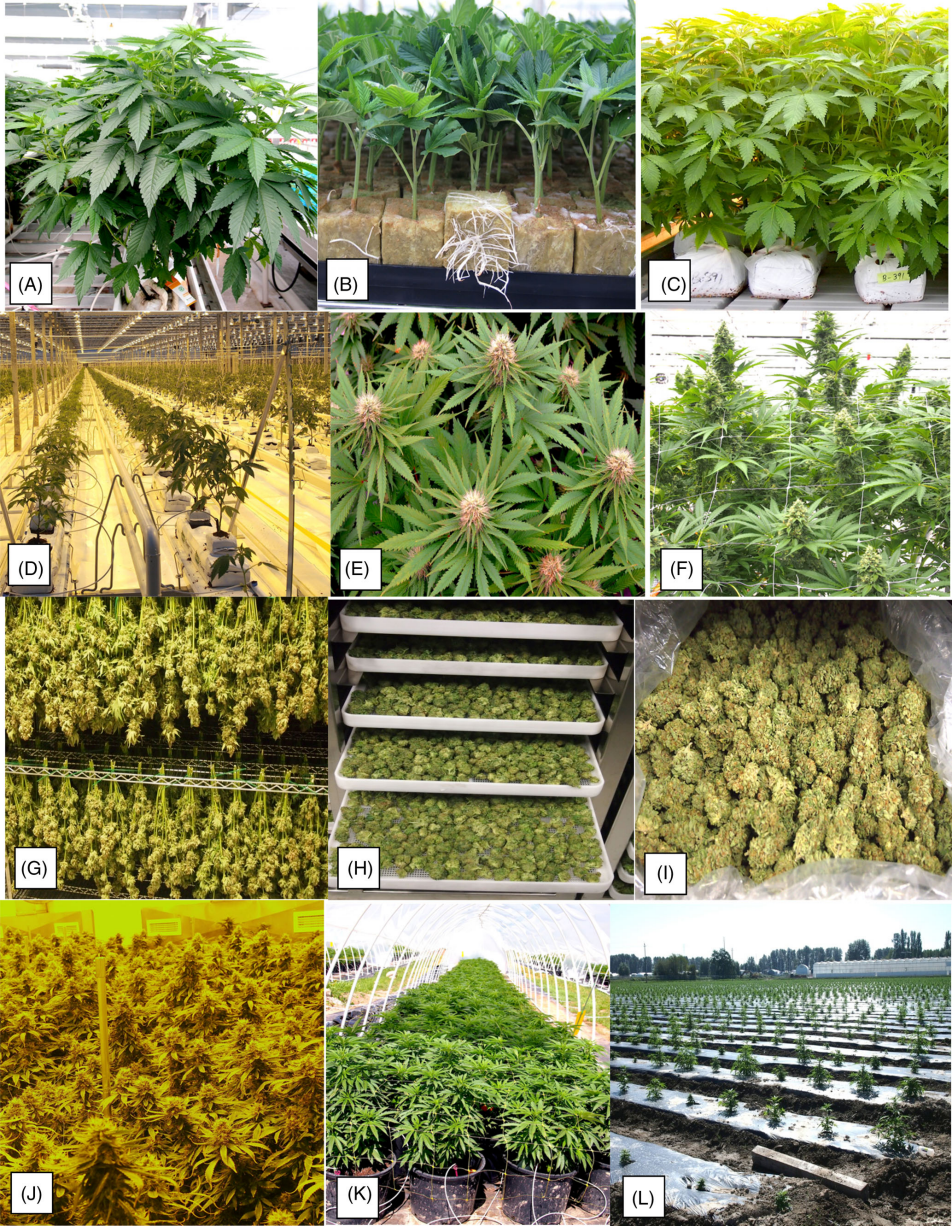
Overview of a cannabis propagation and cultivation scheme. (A) Stock (mother plant) provides a source of cuttings. (B) Rooted cuttings in propagation room. (C) Vegetative plants from cuttings. (D) Plants in flower room. (E) Flowering plants under 12:12 h photoperiod. (F) Plant approaching harvest. (G) Intact inflorescences in the drying room. (H) Detached dried buds. (I) Dried buds at packaging. (J) Indoor production facility. (K) Outdoor production in plastic tunnel. (L) Field production on plastic mulch.

Cultivation of cannabis indoors begins with vegetative propagation from shoots (cuttings) taken from stock (mother) plants (Fig. 1(A)) of a desired genotype (strain or chemovar).^15^, ^16^ Since cannabis is dioecious in its reproductive mechanism,^4^, ^17^ only female plants that bear unfertilized inflorescences are utilized in commercial production. Male plants are of value solely for selective breeding. The term ‘cultivar’ is not applied to cannabis due to the unknown parentage and origin of the thousands of strains believed to be in use. Unique and descriptive names describe these strains, e.g. ‘Billy Jean’, ‘Jack the Ripper’, ‘Girl Scout Cookies’, ‘Powdered Donut’ and ‘Ice Cream Cake’. Some strains may have originated from undocumented breeding experiments under unspecified conditions, with seeds or vegetative cuttings distributed to hobbyists and cannabis enthusiasts.^15^ Molecular and biochemical methods being used to distinguish among cannabis strains show that a high degree of genetic diversity is present,^18^, ^19^, ^20^ a consequence of the out‐breeding nature of this dioecious plant.^4^ These vegetatively propagated strains can harbor undetected or latent fungal or viral pathogens, e.g. *Fusarium oxysporum* causing root and crown rot^21^ and *Hop latent viroid* causing malformation of buds,^22^, ^23^ allowing long‐distance spread of pathogens to occur. The role of seeds in pathogen dispersal has not been extensively studied in cannabis, although a number of fungi present on hemp seed, such as *Alternaria*, can potentially initiate infection of the developing seedling. Fungicide seed treatment options for hemp are currently under investigation.

To induce roots on cuttings, a hormone such as indole‐acetic acid (IAA) is used (Fig. 1(B)); plants are then transferred to a growing substrate, e.g. cocofibre, rockwool or peat/soil, after 2 weeks. These vegetative plants (Fig. 1(C)) receive 16–24 h of continuous supplemental lighting per day. The general plant vigor and health, i.e. absence of disease symptoms, determines the success of the crop and ensuing yield (grams of dry weight of inflorescences harvested per m^2^ of growing space). Four weeks after cuttings are taken, plants are transferred to a ‘flowering room’ (Fig. 1(D)), where the photoperiod is reduced to 12 h of complete darkness. Since cannabis is a short‐day (long‐night) plant, the 12:12 h photoperiod triggers the onset of inflorescence development,^4^ which progresses over an 8‐week period to harvest (Fig. 1(E)). The molecular and physiological basis for the trigger of flowering by reduced photoperiods deserves further attention. At maturity, inflorescences are hand‐harvested and dried by hanging them upside down (Fig. 1(F)) or after mechanical removal of buds from the stem while fresh (Fig. 1(G)). The buds are then dried to approximately 10% moisture (by weight) in specifically designed drying rooms at ambient humidity of 50–55% and temperatures of 17–21 °C over 5 days (Fig. 1(H)).^24^ Diseased inflorescences or drying rooms that do not maintain optimal environmental conditions can result in postharvest mold development, which can reduce product quality and pose potential harm to humans.^25^ Mold management in cannabis products is a major challenge for producers and postharvest losses exceeding 10–15% are not uncommon.

Cultivation of hemp occurs mostly outdoors, with plants initiated directly from seed or occasionally from transplanting of rooted cuttings produced in greenhouses as described above. There is no significant indoor commercial cultivation of hemp other than for propagation. In contrast to cannabis, cultivars of hemp, developed in Canada and Europe for various agronomic traits such as high fibre, high quality seed (grain), and, more recently, cannabidiol (CBD) content, are well characterized. Canadian producers currently have more than 50 cultivars approved for production. The major challenges facing expanding cultivation of hemp are seed quality and pressure from weeds, insects and diseases.

## 3 DISEASE SYMPTOMS

**Figure 2:**
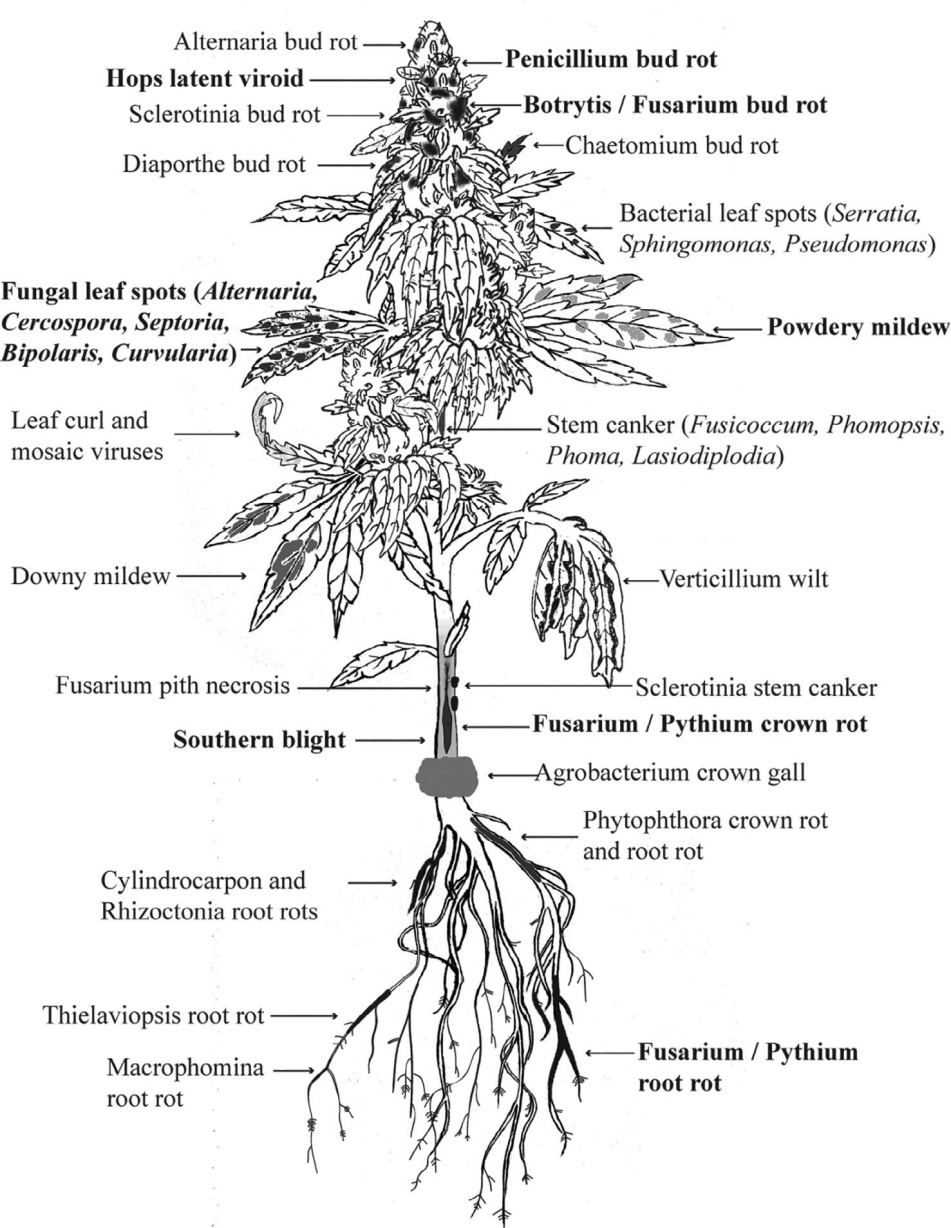
The emerging pathogens on cannabis and hemp plants. Pathogens shown in **bold** are the most damaging.

**Figure 3:**
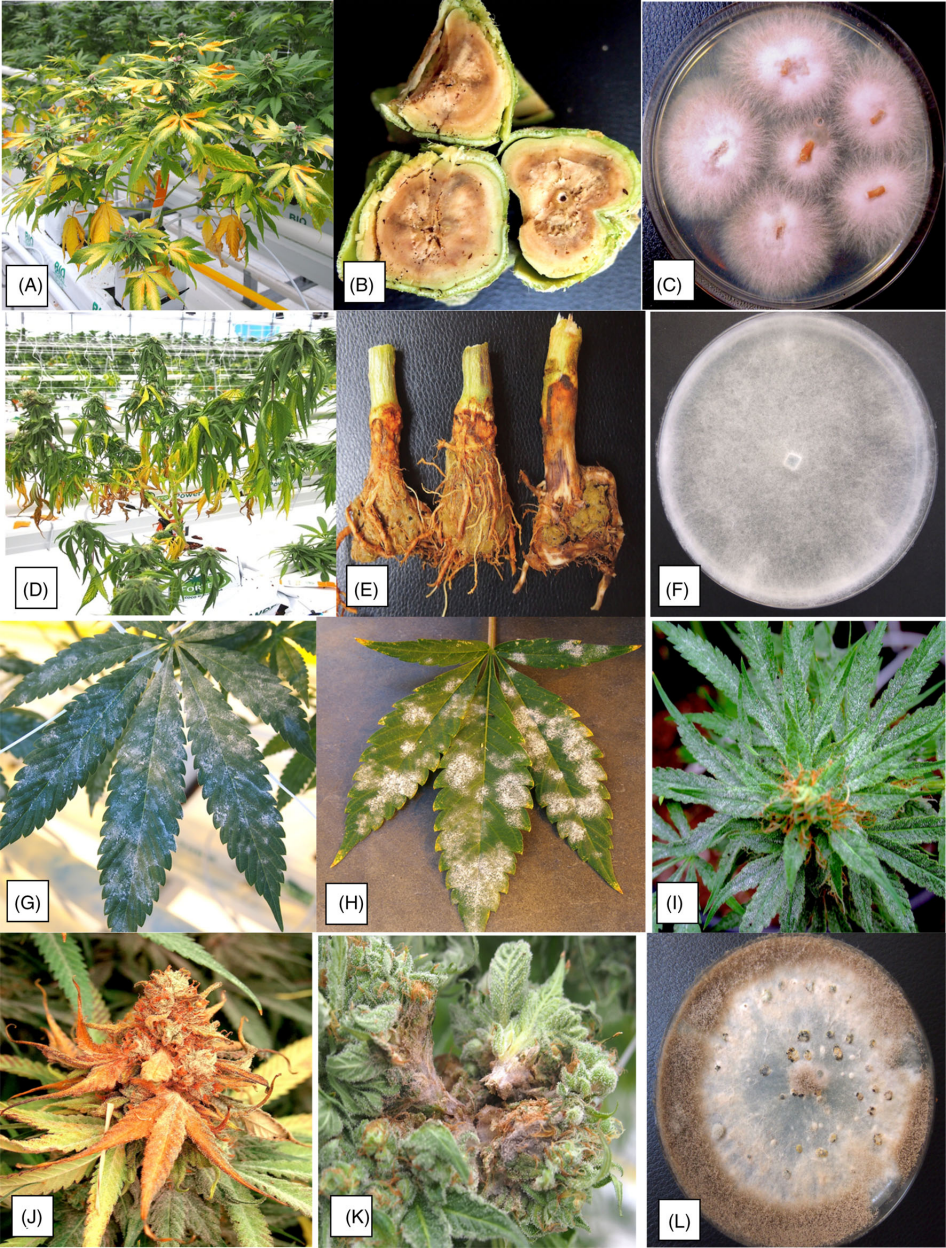
The pathogens that infect cannabis plants cause different symptoms. (A)–(C) *Fusarium* causes yellowing of foliage and internal stem necrosis. (D)–(F) *Pythium* causes wilt and crown necrosis. (G)–(I) *Golovinomyces* causes powdery mildew on leaves and inflorescences. (J)–(L) *Botrytis* causes bud rot. Respective pathogen cultures are shown in (C), (F) and (L).

Generally, the type of plant disease symptoms can provide a preliminary assessment of the causal agent involved.^26^ In cannabis and hemp, descriptions of recently emerging diseases have begun to appear in the published literature (Table S1). The pathogens can be grouped by the tissues they infect: root and crown‐infecting, foliar and stem‐infecting, inflorescence‐infecting, and postharvest pathogens (Fig. 2). Most of the pathogens are fungi and oomycetes, followed by viruses or viroids. Bacterial pathogens are less commonly reported **(**Table S1). The most destructive root pathogens are *Fusarium* and *Pythium* species (Fig. 3(A)–(F)), particularly when infections occur during the rooting phase or vegetative growth. These established infections may progress into the flowering stage, causing stunting and ultimately plant death (Fig. 3(A),(D)). If *Fusarium* and *Pythium* occur concurrently on root and crown tissues, severe symptoms, such as sudden and rapid death of flowering plants, can occur. Losses caused by these two pathogens can be as high as 30%. Disease control methods for root‐infecting pathogens must be implemented early during the production cycle (within the first 2–4 weeks) for effective management. For example, application of biological control agents should be made during this phase to allow effective root colonization and pathogen competition, as discussed in section 8.6. The foliar‐infecting pathogens of cannabis and hemp include powdery mildew (Fig. 3(G)–(I)), while hemp cultivated outdoors can be severely affected by several leaf‐spotting fungi, as well as a number of recently identified viruses^27^, ^28^ that have not yet been diagnosed on indoor cultivated cannabis plants (Table S1). The stem‐infecting pathogens are varied and diverse in the symptoms they produce and occur both indoors and outdoors (Table S1), although outdoor grown plants have a higher prevalence of these diseases as they are difficult to manage. Disease control measures for these pathogens rely on prevention of initial infection (exclusion) and reducing subsequent spread of secondary inoculum (spores). The inflorescence‐infecting pathogens are the most damaging to a crop as they directly infect and destroy the buds (Fig. 3(J)–(L)), causing losses of up to 20%. These infections can also lead to significant additional postharvest losses. The most damaging fungi are *Botryis* and *Fusarium* species (Table S1), as well as a number of other fungi that colonize foliar and flower tissues, including *Penicillium* and *Golovinomyces* species. These fungi produce large numbers of spores to ensure spread (Fig. S1). Recently observed fungi that can cause bud rot include species of *Diaporthe* and *Sclerotinia* (Table S1). Hop latent viroid can also cause ‘dudding’ of the buds, which are essentially destroyed as a result of infection.^22^, ^23^ The bud‐infecting fungal pathogens are the most challenging to manage due to a lack of registered fungicides, a lack of information on the sources of inoculum and when infection occurs, and a small suite of biorationale products for use. The extensive development of fungi such as *Fusarium* and *Penicillium* within the inflorescences can also lead to mycotoxin accumulation in the tissues,^29^, ^30^, ^31^, ^32^, ^33^ potentially posing additional health concerns for consumers. This aspect requires further impact assessment by government regulators in Canada and the USA.

## 4 PATHOGEN IDENTIFICATION

Fungal and oomycete pathogen identification to genus level can be achieved using morphological criteria followed by species identification by molecular methods. For cannabis and hemp pathogen identification, the most widely used method is the polymerase chain reaction (PCR) of the ribosomal DNA region that includes the internal transcribed spacer (ITS) and intergeneric spacer regions (IGS)^34^, ^35^, ^36^, ^37^ (Fig. S2). PCR was used to identify most of the species listed in Table S1. In addition, the elongation factor 1 (EF‐1) region was used to discriminate among *Fusarium* species.^21^, ^38^ For *Golovinomyces* causing powdery mildew and *Botrytis* species causing bud rot, additional molecular markers are required to differentiate between species. In the latter, the glyceraldehde‐3‐phosphate dehydrogenase gene (G3PDH) and the heat shock protein 60 gene (HSP60) were used.^39^, ^40^ For the powdery mildew pathogen *Golovinomyces*, however, the ITS region was insufficient to distinguish among species.^41^ Additional molecular markers are needed to conclusively confirm or amalgamate the three species currently reported to cause powdery mildew on cannabis and hemp (Table S1). Following identification, demonstration of pathogenicity on cannabis and hemp plants is an essential requirement – reports of presence or recovery of fungi from these tissues is insufficient, since many saprophytes are found on cannabis and hemp plants. Therefore, fulfillment of Koch's postulates^26^ is essential. Deposition of DNA sequences in a repository such as GenBank will also allow researchers to compare their findings with others. Subspecies or *formae specialis* designations, e.g. f.sp. *cannabis* or *cannabina* for pathogens recovered from cannabis or hemp plants, is discouraged until host range studies and molecular confirmations support these designations. The host of origin is insufficient to imply specific host preference since most of the pathogens affecting cannabis and hemp are known to have wide host ranges (Table S1).

Bacterial plant pathogens have also been identified based on PCR methods that utilize the 16S region of ribosomal RNA (rRNA) in addition to other methods.^35^, ^42^, ^43^ To date, recent reports of bacterial pathogens affecting cannabis or hemp plants and the symptoms they cause are surprisingly few (Table S1); most appear to be saprophytes and incidental/secondary contaminants.^7^, ^9^, ^35^, ^44^, ^45^ Whether or not this is due to innate resistance in the plant or because environmental conditions have not supported bacterial infection or spread remains to be determined. It is likely that members of the genera *Pseudomonas*, *Xanthomonas* and *Pectobacterium* will be the most commonly occurring bacterial pathogens in addition to those reported already. Viral and viroid pathogens of cannabis and hemp have been identified by molecular analysis that involves RT‐PCR of RNA templates.^22^, ^23^ In addition, next‐generation sequencing (NGS) approaches are revealing the presence of previously unreported viruses in hemp.^27^ The number of virus diseases occurring on hemp is steadily increasing as more research identifies new symptomology and spread of these pathogens from other host species.^27^ There are few viruses currently reported on cannabis but this is likely due to a lack of research efforts. Earlier reports of viral pathogens reported to infect *C. sativa* ^11^, ^13^ need to be reassessed using modern molecular diagnostic and NGS approaches. Management of viral diseases will be a challenge in a crop that is primarily propagated through vegetative means, since viruses are known to be transmitted from cuttings as well as through seed. Planting materials indexed and certified to be virus‐ or viroid‐free will be the key to minimize spread. The role of insect vectors in dissemination of viruses to cannabis and hemp plants will require further research. Many of the recently reported viruses on hemp are reported to be seed and vector transmitted,^27^, ^46^ adding an extra layer of complexity with regard to disease management.

## 5 QUANTIFICATION OF MICROBES ON INFLORESCENCES

Government regulations require quantification of total yeast, mold, bacteria and other pathogens in dried cannabis samples to ensure they do not pose potential risks to human health. Certified laboratories perform a set of microbial isolations to enumerate total culturable yeast and mold (TCYM) and total bacteria, as well as coliforms, expressed as colony‐forming units (cfu).^7^ These certifications are required by provincial (state) and federal regulatory agencies in Canada and the USA, as well as in Europe,^10^ Israel^47^ and other countries. The assessment methods can be vastly different and require standardization to ensure consistency and reproducibility, as well as to provide recovery rates and validation based on known standard samples. There is ongoing debate on whether culture methods provide the most representative assessment of microbial load present on cannabis buds.^7^, ^35^ Many of these methods are adapted from the food manufacturing industry, yet the microbial flora on plants are vastly different. The diversity of microbes that are present on fresh inflorescences of cannabis can be ascertained through sampling methods (Fig. S3). Many of these microbes can survive the postharvest drying phase but the relationship of microbial loads on buds preharvest to those postharvest has not been established. Research to develop models to predict final cfu/g from preharvest assessments would be useful. The fungal species found on dried cannabis buds in commercial production are diverse and exceed 35 different species.^25^ Other researchers have described the yeast and bacterial loads.^7^, ^35^, ^48^ There are recommendations that molecular approaches utilizing quantitative PCR (q‐PCR) would be more informative for microbial determination on cannabis inflorescences compared to plating assays.^7^, ^35^ Regulatory agencies have not provided a framework of recommended methods or best practices for quantification of these microbes and thus the variability in findings will persist. This can make interpretation of QA results from commercial laboratories challenging for cannabis producers. Instances of the same batch of dried flowers yielding different mold counts from two different laboratories indicate that regulated and standardized procedures are required. The total cultural assessment data also do not identify the actual yeast or mold species present, which can be quite varied.^25^ For example, a potential beneficial biological control agent such as *Trichoderma harzianum*, if it is present in high numbers on buds, may cause a product to fail to meet the limit requirements. Other commercial products containing the fermentation end‐products from *Lactobacillus* spp. may result in higher yeast and mold counts after application, perhaps as a result of nutrients and other secondary products causing a ‘flush’ of resident microbes to grow. These changes in microbial loads from the application of bio‐rationale treatments need to be monitored to assess their impact on product quality. Currently, such studies are lacking. Also needed are studies to assess the changes in microbial flora during inflorescence development up to harvest.

## 6 RESIDENT MICROBES (ENDOPHYTES): BENEFICIAL OR DETRIMENTAL?

Most plant species harbor a suite of microbes (fungal, yeast, bacterial and actinomycete species) present internally that survive tissue surface‐sterilization methods used to eliminate external contaminants. These endophytes have generated considerable interest in basic and applied research studies to elucidate their roles within the plant.^49^ Some endophytes are saprophytic, i.e. live on dead tissues, some are pathogenic, while others may provide beneficial outcomes in plants. Reports of enhanced tolerance to insect pests and plant pathogens, tolerance to drought stress, and enhanced nutrient uptake are examples of beneficial effects.^50^, ^51^, ^52^ Negative effects are less frequently reported, perhaps because researchers attribute less importance to such data in the search for beneficial outcomes. Microbial residents within the plant can have multiple roles, depending on environmental conditions, growing conditions and host genotype. For example, *Aspergillus* and *Penicillium* species are amongst the endophytes previously described from cannabis plants.^53^, ^54^ However, these fungi can pose potential health risks to humans and are not beneficial. A diverse group of fungal and bacterial species were identified in hemp and cannabis plants by direct plating methods and molecular analyses.^35^, ^44^ While many of them were proposed to benefit the plant,^44^, ^45^, ^53^, ^54^, ^55^ establishing effects on the growth of cannabis plants requires rigorous experimentation, similar to that required for proof of pathogenicity of presumed pathogens. Proposed beneficial roles of endophytes in cannabis and hemp plants that appear in the literature, therefore, should be interpreted cautiously until evidence has been obtained through experimentation. For example, there is no evidence to suggest that endophytes can alter the secondary metabolism and cannabinoid profiles in cannabis^45^, ^55^ or provide protection against pathogens.^53^, ^54^ The metabolic pathways and genes that lead to cannabinoid production are complex^1^, ^3^, ^56^, ^57^ and evidence of transcriptional or translational regulation by endophytes is lacking. Comeau *et al*.^58^ observed no correlation between the microbiome composition, including those in root tissues, and cannabinoid levels produced in cannabis strains, which is genetically determined. The utility of molecular approaches to elucidate the microbiome (the totality of microbial constituents associated with cannabis plant tissues)^9^, ^31^ should shed some light on the potential roles of these internalized microbes. In a recent study, Barnett *et al*.^59^ showed significantly disparate populations of microbes were associated with different tissues of hemp plants. Therefore, attempting to correlate functional aspects of cannabis plant growth with presumed roles of a range of microbial populations that are continuously changing will prove to be a challenging endeavor.^59^ Recovery of specific microbes and their reintroduction into the cannabis growing environment should identify more concrete functional roles (both positive and negative) of endophytes. Recently described metagenomic‐based approaches, which identify resident microbes on cannabis or hemp tissues using sequence data and then associate presumed functions to them based on prior literature reports,^58^, ^59^, ^60^ will not provide the much‐needed cause‐and‐effect determination or reveal insights into the importance and roles of microbiomes in cannabis. Changes in the microbiome composition with maturation of cannabis inflorescences, for example, would provide more insightful and practical information.

The tissues harboring endophytes in cannabis plants include the pith and surrounding parenchyma cells (Fig. S3), which can support growth of *Penicillium* species in large numbers. The microbiome of pith tissues has not been previously studied. The pith parenchyma cells disintegrate in a manner similar to programmed cell death,^38^ leaving behind layers of dead cells suitable for microbial colonization. Fungi recovered from pith tissues include species of *Chaetomium*, *Trichoderma*, *Cladosporium* and others.^61^ These species possess strong cellulolytic enzyme activities which are required for growth on cell walls (Fig. S3). Their origins may be from the growing substrate, e.g. cocofibre, or the surrounding environment.^61^ In addition, leaves, stems, petioles and flowers of cannabis and hemp are reported to harbor a range of fungi and yeasts.^44^, ^59^ In other plants, internalized microbes may be found in meristematic regions such as root and shoot tips.^49^ The mechanized process of removing cannabis flower buds from stems after harvest disrupts the stem tissues, which can release spores of endophytes, resulting in a build‐up of air‐borne propagules.^61^ Up to 17 species of *Penicillium* were identified on commercially dried cannabis buds.^25^ These endophytes can also be problematic during tissue culture of cannabis as surface‐sterilization methods do not eliminate many of them and they can continue to grow on nutrient‐rich media and inhibit explant growth. Endophytes have been shown to be problematic in tissue culture experiments with other plant species.^62^ The limited research to date suggests that endophytes in cannabis and hemp plants pose problems and few have yet to be shown to provide benefits, hence additional research is important to clarify their roles.

## 7 EPIDEMIOLOGY AND PATHOGEN SPREAD

### 7.1 Indoor environments for cannabis

The spread of plant pathogens is a critical component of disease development, allowing inoculum to be disseminated from one plant to another or from one location or region to another. In fungal pathogens, production of large numbers of spores from diseased tissues ensures rapid and widespread movement. Spores of powdery mildew and *Botrytis* species are produced on conidiophores (Fig. S1) and released into the air to spread over long distances, while those of *F. oxysporum* can be disseminated through air, water or on plant material.^21^ Spores of *Penicillium* species are also present in most indoor environments, and on decaying plant materials and soil, and are spread readily in the air both indoors and outdoors.^33^ There are seasonal differences in the populations of spores of fungal plant pathogens and saprophytic molds,^63^ and greater numbers are found in the autumn season, which would be particularly important for outdoor grown cannabis and hemp. In indoor growing environments, the introduction of diseased plant materials as cuttings or stock plants can result in the spread of pathogens such as powdery mildew, *Fusarium* spp., *Hop latent viroid* and potentially other pathogens. Initial inoculum of root‐infecting pathogens and mold contaminants can also be introduced through infested growing medium (cocofibre, soil) if it has not been adequately sterilized.^61^ Once a pathogen is introduced within a growing facility, e.g. a greenhouse, inoculum of pathogens such as *Pythium* and *Fusarium* can spread in unfiltered recirculated water, in air and potentially by workers.^64^ Seed‐borne dissemination is also another means by which fungal and viral pathogens can be introduced into a growing facility or region.^65^ The importance of seed‐borne pathogens in cannabis and hemp requires further research.

### 7.2 Field environments for cannabis and hemp

**Figure 4:**
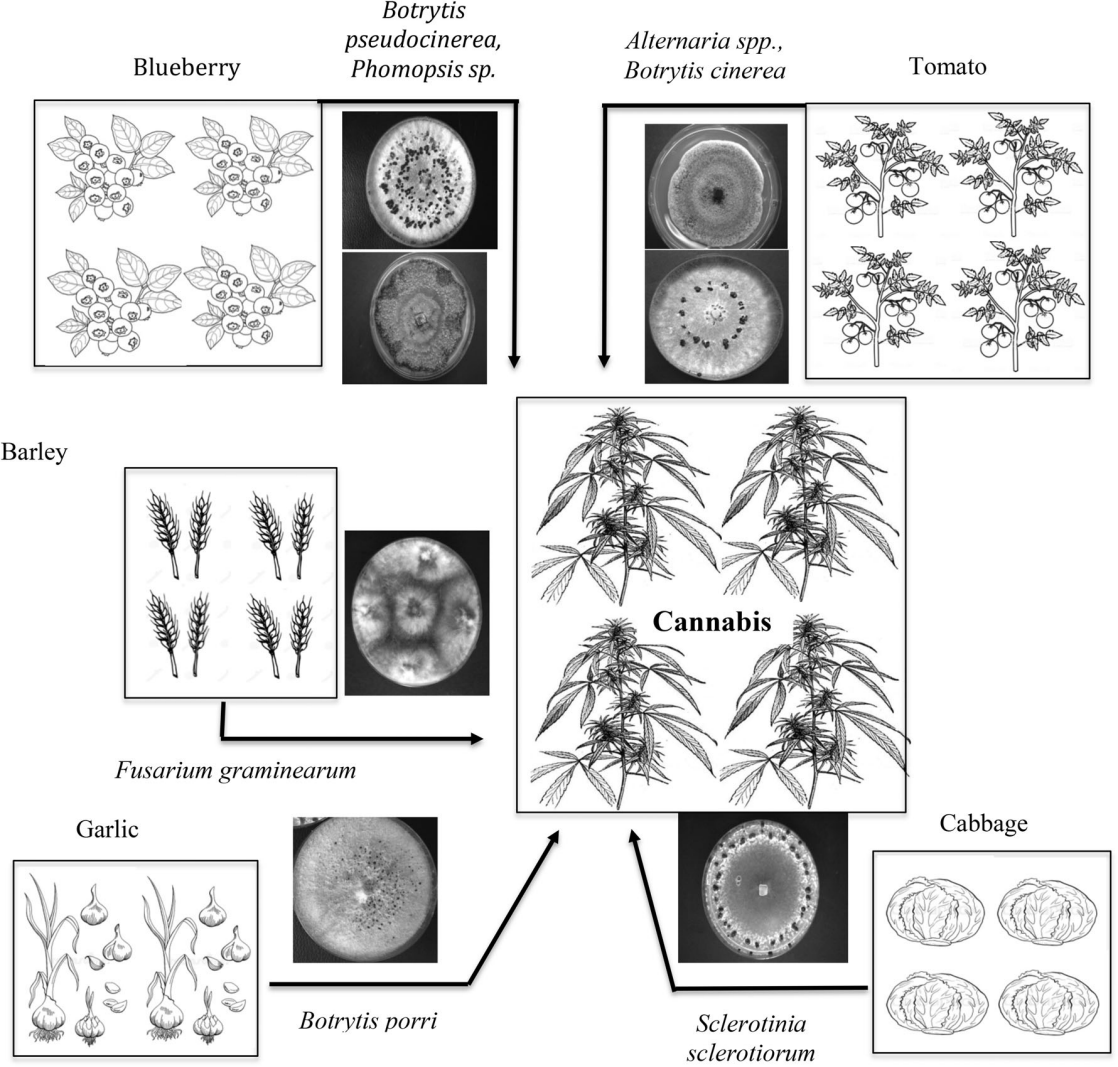
Pathogen inoculum sources for cannabis and hemp plants can originate from neighboring fields cropped to blueberry, tomato, barley, garlic and cabbage, which release inoculum to cause infection.

Under field conditions, the extent of pathogen spread is dependent on environmental conditions such as wind and rainfall, as well as proximity to neighboring fields in which susceptible hosts are grown. For field or plastic tunnel cultivation, spread of inoculum from adjacent fields can result in subsequent disease development (Fig. 4). For example, spread of spores of *Phomopsis* from *Diaporthe eres*‐infected stem cankers and of *Botrytis pseudocinerea* from blueberry plants showed recovery of both pathogens from bud rot‐infected cannabis inflorescences (Fig. 4). Similarly, tomato fields with grey mold due to *Botrytis cinerea* and cabbage fields with *Sclerotinia* stem canker gave rise to bud rot caused by these two pathogens in adjacent fields. In all cases, neighboring fields were within 100–200 m from cannabis fields. These observations indicate that cannabis inflorescences are highly susceptible to infection by fungi that may originate from neighboring fields with diseased plants. Similarly, hemp leaves and stems are susceptible to many leaf‐spotting fungi as well as a range of canker and die‐back pathogens (Table S1), some of which can devastate the crop. The spread of pathogens from hop plants to cannabis plants has also been reported for powdery mildew and *Hop latent viroid*.^22^, ^23^, ^66^, ^67^ Hemp and cannabis plants are susceptible to both the hop powdery mildew pathogen (*Podosphaeria macularis*) as well as the predominant cannabis powdery mildew pathogen (*Golovinomyces* spp.).^67^ The spread of pathogens from adjacent unrelated crop plants, and from hop plants, through release of inoculum, as well as spread by insect vectors of viruses, is an unexpected consequence of the expanding cannabis and hemp industries in Canada and the USA. Additionally, planting hemp or cannabis in fields previously cropped to other hosts with residual inoculum can result in disease development. For example, fields under pasture followed by planting to hemp resulted in crown rot due to *Fusarium avenaceum*, *F. graminearum* and *F. tricinctum* (Table S1). Lettuce fields with *Sclerotinia* infection resulted in white mold development in a subsequent crop of hemp.^68^ Lastly, inoculum of *Sclerotium rolfsii* in fields planted to peanuts or vegetable crops can cause southern blight on hemp the following season in many US southern states, especially under excessively hot conditions (Table S1). Knowledge of previous cropping history prior to selection of a site for cannabis or hemp cultivation should indicate the potential for disease occurrence. Monitoring cultivated crops or vegetation adjacent to hemp or cannabis fields may demonstrate the risk of inoculum spread, particularly if insect vectors are involved. Further research is needed to determine the types of crops that could be used in rotation with hemp or cannabis to minimize pathogen inoculum build‐up and carry‐over.

## 8 DISEASE MITIGATION APPROACHES

Mitigation of disease development requires disruption of the disease cycle, beginning with prevention of inoculum introduction, preventing infection or symptom development, reducing secondary inoculum production and spread, and reducing survival of the pathogen. These approaches are summarized in Table 1 and will be discussed with reference to specific pathogens that can be mitigated to prevent infection of cannabis or hemp plants.

### 8.1 Prevention of inoculum introduction

The prevention of inoculum introduction is critical for producers starting a new crop. This can be ensured through testing of stock plants for the presence of pathogens prior to vegetative cuttings being taken, disinfesting seeds and equipment to eradicate pathogen propagules, ensuring that planting materials, substrates and water sources are free from pathogen inoculum, and ensuring that cannabis and hemp crops are not planted in fields with a prior disease outbreak in the preceding crop, or adjacent to other crops that could be a source of inoculum. Methods for testing of plants to ensure they and the planting materials are free from pathogens are widely available and several commercial laboratories can provide detection services. PCR methods for detection of many species reported to infect cannabis and hemp, including *Fusarium* and *Pythium*,^21^, ^69^, ^70^ as well as *Golovinomyces* species,^41^, ^71^ have been described. *Hop latent viroid* can be detected using molecular techniques.^22^, ^23^, ^72^ Methods for detecting seed‐borne pathogens on cannabis or hemp seeds can be adapted from the vast literature on seed‐borne pathogens.^65^, ^73^ Since there is currently no certification program for cannabis or hemp in Canada, licensed producers cannot be guaranteed disease‐free planting materials. As a result, spread of *Fusarium* through cuttings provided by propagators, for example, is not uncommon, resulting in introduction of the pathogen into new areas. The powdery and downy mildew pathogens, and *Hop latent viroid*, can also be introduced on diseased cuttings, as these obligate pathogens require a living host for reproduction and survival. Many producers rely on propagators that may not be performing due diligence in ensuring that materials sold for distribution are certified pathogen‐free. The development of tissue culture methods for cannabis and hemp has shown some success with regard to regeneration of shoots from nodal explants for micropropagation.^74^, ^75^ While several commercial companies are now producing plantlets, this approach needs to be augmented with screening for pathogens to provide disease‐free planting materials. Currently, there are no certified disease‐free plants that have been derived through tissue culture. The importance of virus‐free planting materials and early detection for management of viral and viroid pathogens has been emphasized.^27^

### 8.2 Prevention of infection/symptom development

Fungicides that target fungal spore germination to prevent initial infection or symptom development are widely available for most agricultural crops.^26^ Most are applied at multiple times during the season and safety data have been generated. For cannabis or hemp, however, no fungicides are currently registered for use although vaporized sulfur can be used effectively to minimize establishment of powdery mildew under greenhouse conditions. Since fungicide applications are common in most food crops, the development of similar safety data allowing their use on cannabis plants during the early propagative stages, on stock plants and as seed treatments to reduce the development of pathogens such as *Fusarium* and *Pythium* is warranted. Fungicides with active ingredients that include metalaxyl, strobilurins, fludioxonil, fluopyram and pyrimethanil can provide effective control of the most important emerging pathogens (*Pythium*, *Fusarium*, *Botrytis* and powdery mildew). Restrictions on application times and monitoring of residues will provide the necessary safety measures for consumers. Applications of potassium bicarbonate (MilStop) sprays at weekly intervals were shown to reduce powdery mildew development on cannabis plants^76^ (Fig. S4). The application of fungal or bacterial biological control agents to reduce initial infection is described in detail in section 8.6. Application of a plant extract (Regalia Maxx) from the noxious weed giant knotweed (*Reynoutria sachalinensis*) also reduced powdery mildew development on cannabis (Fig. S4). This product is registered for use on cannabis in Canada and is reported to induce disease resistance when applied to a range of plant species prior to the initiation of infection;^76^ it has less activity when used post infection. The knotweed extract has been reported to reduce powdery mildew development on cucumber, tomato, squash and wasabi plants.^76^ The potential for induced resistance mechanisms in treated cannabis plants remains to be determined. The application of biological control agents to reduce infection by *Fusarium* on cannabis cuttings is described in section 8.6. These results indicate that there are several approaches that can be used to reduce infection if used at the appropriate time.

### 8.3 Reducing secondary inoculum production

Reducing sporulation of pathogens such as powdery mildew can be achieved by applications of potassium bicarbonate as well as by biological control formulations that contain *Bacillus* sp., which are known to produce a range of antifungal antibiotic compounds.^77^, ^78^ In addition, removal of infected leaves (deleafing) is conducted by some producers to minimize secondary inoculum production and spread of powdery mildew. Removal of *Botrytis*‐infected flower buds can also slow down the rate of spread of the pathogen by minimizing inoculum production. However, given the rapid rate at which these pathogens can reproduce and the vast number of propagules produced (Fig. S1), reduction of secondary inoculum production is not always effective at reducing the rate of disease spread. It is difficult to demonstrate if specific treatments that reduce secondary inoculum production by root‐infecting pathogens can potentially reduce spread. Treatments of recirculating nutrient solution have been shown to reduce inoculum levels of *Pythium*.^64^

### 8.4 Reducing pathogen survival

Approaches to reduce survival of pathogen propagules should emphasize sanitation measures, such as removal and destruction of all plant materials and debris that could contain inoculum of pathogens such as *Fusarium*, *Botrytis* and *Sclerotinia*, and disinfecting surfaces used during cannabis cultivation, e.g. propagation benches, with hydrogen peroxide, UV sterilization and other disinfectants, e.g. didecyl dimethyl ammonium chloride, to reduce inoculum carry‐over. Treatment of recirculated water may be required to ensure pathogen inoculum is not reintroduced into greenhouse production systems.^79^ The addition of chlorine can reduce carry‐over of inoculum of *Pythium* and *Fusarium*.^64^ Under field conditions, inoculum survival can be reduced through implementation of sanitation measures such as removal and destruction of diseased plants, burial of diseased tissues deep in the soil and crop rotation. Application of microbial antagonists to reduce pathogen survival has had some success on other crops^80^ but requires a longer‐term time frame for success.

### 8.5 Managing pathogens that infect the inflorescences

The greatest challenge remains in dealing with pathogens that infect cannabis inflorescences (Table S1) since economic losses from tissue destruction and a build‐up of colony‐forming units pre and post harvest can be as high as 20%. Research on this topic is limited by government regulations in Canada which restrict the cultivation of flowering cannabis plants to producers with approved licenses; researchers have limited capacity to grow such plants. Indoor climate management to provide dry conditions is recommended to reduce Botrytis bud rot, since reduced relative humidity and moisture deposition on the inflorescences can reduce spore germination and infection.^81^ Under field conditions, Botrytis bud rot can be a devastating disease under cool and wet weather on cannabis and hemp^25^, ^39^; cultivation under warm dry conditions would alleviate disease pressure. Cannabis strains have noticeable differences in Botrytis bud rot susceptibility: those that produce large tightly packed inflorescences develop more disease that those with smaller and loosely arranged inflorescences. In the latter, improved air movement within the canopy is assumed to be the underlying reason. It may be useful to evaluate pruning or deleafing methods to enhance air circulation, similar to that demonstrated for grapevines and Botrytis bunch rot reduction.^82^ Removal and destruction of diseased inflorescences and early harvest are currently practiced by licensed producers to reduce the potential for spore production and pathogen spread. These physical methods can minimize disease outbreaks but are labor‐intensive. The application of fungicidal or fungistatic compounds is restricted to the use of vaporized sulphur for powdery mildew control and potassium bicarbonate and hydrogen peroxide applications for powdery mildew and potentially *Botrytis* reduction. Concerns over possible fungicide residue carry‐over in the inflorescence tissues used for medical or recreational purposes has limited the registration of synthetic fungicides. A reassessment of the utility of fungicides during cannabis production to enable producers to manage diseases is warranted.

### 8.6 Biological control agents for disease management

The application of biological control agents to reduce disease development on cannabis and hemp offers great potential in light of the limited availability of synthetic fungicides. Although several biological control agents are registered for use on cannabis in Canada to manage diseases, comparative efficacy data are lacking. Products based on *Trichoderma harzianum*, *T. asperellum* and *Gliocladium catenulatum*, as well as *Bacillus subtilis* strain QST 713 and *Bacillus amyloliquefaciens* strain F727 are being evaluated in our laboratory. The effects on three diseases are under investigation: *Fusarium* damping off on cannabis cuttings, powdery mildew on foliage and *Botrytis* infection of the inflorescence. Biocontrol activity against *Fusarium* on cannabis cuttings was assessed by applying products at recommended rates 48 h prior to pathogen challenge. Disease development in hydroponic cultivation after 2 weeks is shown in Fig. S5(A,C,E). All microbes demonstrated efficacy against this pathogen compared to the control (Fig. S5(F)). The internal colonization of treated stem cuttings by *Trichoderma* and *Gliocladium* after surface‐sterilization and plating onto agar medium is shown in Fig. S5(B,D,F). *Fusarium*‐treated cuttings gave rise to only colonies of the pathogen (Fig. S5(G)). These preliminary results show that endophytic colonization by *G. catenulatum* and *T. asperelllum* can occur and is an important component of the biocontrol activity of these two microbes^83^, ^84^, ^85^ and this is likely to be taking place in cannabis stems.

For powdery mildew control, weekly applications of *B. subtilis* strain QST 713 were as effective as potassium bicarbonate and knotweed extract for disease suppression (Fig. S4). In contrast, neither *Streptomyces lydicus* strain WYEC 43 or hydrogen peroxide had a significant effect.^76^ For *Botrytis* management, *T. asperellum* and *B. amyloliquefaciens* were tested *in vitro* and both significantly inhibited pathogen growth (Fig. S6). When they were applied to detached inflorescences 48 h prior to pathogen inoculation, they visibly reduced pathogen development after incubation under high humidity for 7 days (Fig. S6). These preliminary results are encouraging, prompting additional studies to determine the time of application and rates required on whole plants. Can applications of biocontrol products to inflorescences at predetermined times prior to harvest allow sufficient colony‐forming units to inhibit the pathogen but which do not cause products to fail to pass regulatory requirements? Further studies on survival and modes of action are needed, recognizing that the extensive published literature on biocontrol mechanisms^77^, ^78^, ^86^, ^87^, ^88^ are likely to be applicable to cannabis.

### 8.7 Efficacy of ultraviolet light and irradiation for disease management

#### 8.7.1 Preharvest irradiation

Irradiation of foliage with UV‐C at 3–6 mJ cm^−2^ for 3–5 s daily for 28 days significantly reduced powdery mildew development (Fig. S4). The use of ultraviolet light (UV‐C and UV‐B) to reduce pathogen and mold development has been demonstrated for several crops, including for powdery mildew management.^76^ The efficacy of UV light in managing diseases is due to its direct germicidal activity as well as its indirect ability to induce defense responses in plants, including increased levels of phenolic compounds and pathogenesis‐related proteins.^76^ Whether UV‐C can minimize mold growth when applied to inflorescences during the plant growth cycle, without causing damage to tissues that may inadvertently increase mold levels, requires further study. It is also not known what effect preharvest UV‐C treatment may have on cannabinoid levels in the inflorescences.

#### 8.7.2 Postharvest irradiation

Irradiation is an approach that is also widely used to minimize growth of molds on stored food products, including dried herbs and fruits.^89^, ^90^, ^91^ On cannabis, irradiation includes the use of gamma rays and electrobeam radiation,^47^, ^92^ as well as cold plasma treatment.^47^ The use of electrobeam irradiation on dried cannabis buds is permitted in Canada to reduce final mold levels. Populations of *Penicillium* spp. and *Botrytis* were significantly reduced following this treatment.^25^, ^47^ Organic producers have to resort to other less well studied methods to reduce overall mold counts which may include postharvest exposure to ozone as irradiation is not permitted.

### 8.8 The search for genetic resistance to pathogens

The genetic background of *C. sativa* includes land races and genotypes whose origins are geographically very diverse.^19^, ^20^, ^57^ This suggests there should be sufficient genetic diversity to identify specific genotypes (strains) with resistance to important pathogens such as *Fusarium*, powdery mildew, *Botrytis*, and viruses and viroids. Screening methods need to be developed to accurately identify such sources, followed by molecular characterization of the underlying biochemical mechanisms and regulatory genes. Among currently grown genotypes, testing of 13 strains revealed none had resistance to *F. oxysporum*,^21^ while four out of 12 strains showed resistance to powdery mildew.^76^ Combined resistance to multiple pathogens, e.g. *Fusarium*, *Botrytis* and *Golovinomyces*, has not yet been achieved through conventional breeding. Studies conducted on other crop species have identified the mechanisms of resistance to *Fusarium* and powdery mildew^93^, ^94^, ^95^, ^96^, ^97^, ^98^ and they should be valuable in directing further research into the underlying disease resistance responses in cannabis plants. The search for resistance to viral pathogens is in its infancy.^27^ Differences in susceptibility to *Botrytis* infection have also been observed that may be related to canopy architecture and inflorescence size and composition as it affects microclimate and relative humidity around the infection sites. Whether or not the secretory products of trichomes^99^ can influence the development of pathogens and molds remains to be determined. The transgenic expression of an antifungal protein in trichomes reduced development of *Botrytis* on *Arabidopsis*.^100^ The possibility of genetic engineering of *C. sativa* to enhance resistance to pathogens^57^, ^101^, ^102^ and alter other quality attributes^1^ is under investigation in several laboratories. This species is amenable to genetic engineering but has proven to be challenging with regard to regenerating fully developed transgenic plants for further testing.^103^, ^104^ The potential to develop transgenic cannabis plants, similar to those in a vast range of other plant species, should be explored.

## 9 CONCLUSIONS AND FUTURE PERSPECTIVES

Over the past 4 years, a large number of fungal, viral, bacterial and nematode pathogens have been reported to cause diseases on cannabis and hemp crops in North America. This review has attempted to summarize these diseases and discuss mitigation approaches utilizing a number of strategies. The recent lifting of restrictions on the cultivation of these crops in North America will encourage peer‐reviewed research to be conducted. The implementation of certified pathogen‐free planting material is an important first step, followed by the utility of biological control agents, which still require research to determine their comparative efficacies and modes of action. The registration of selective fungicides to combat pathogens during the propagative stage should be addressed, with zero residue limits imposed prior to harvest of the final product. The current ‘no fungicide’ enforcement does not address the considerable losses that fungal and oomycete pathogens are causing to the cannabis and hemp industries. Research into the roles that microbial endophytes may play should provide unbiased assessments of their function, as well as of the total microbiome, in cannabis and hemp biology. Methods to accurately assess populations of mold contaminants to distinguish those that could potentially be harmful from those that may be beneficial will be challenging to develop but must be pursued. While postharvest irradiation effectively minimizes mold contaminants, it increases the cost of production and any possible effects on the organoleptic properties of the product need to be assessed. Other treatment options need to be explored for organic producers. Finally, identifying potential sources of resistance to the most challenging pathogens should become a long‐term priority. The surge in cannabis and hemp cultivation is providing a multitude of opportunities for collaboration between academic and industrial researchers to solve emerging disease problems, and with governmental agencies to ensure that consumer safety always remains a top priority.

I am gratified by the recent scientific discoveries that have been published, and look forward to those that have yet to be made. I feel privileged to work on cannabis, an intriguing ‘plant of a thousand and one molecules’.^1^

## CONFLICT OF INTEREST

The author has no conflict of interest to declare.

## Supporting information

**Table S1**. Emerging pathogens reported on cannabis and hemp plants during 2017–2020.

**Figure S1**. Spore production by the most important pathogens affecting cannabis and hemp production: (A) *Fusarium oxysporum*, (B) *Botrytis cinerea*, (C) *Golovinomyces* species, (D) *Penicillium* species, (E, F) *Aspergillus* sp. (A)–(D) are reproduced from the *Canadian Journal of Plant Pathology* by permission from the Canadian Phytopathological Society.

**Figure S2**. Gel electrophoresis of PCR products after amplification of DNA with primers for the internal transcribed spacer region (ITS1‐5.8S‐ITS2) of rDNA. Lane L, molecular weight standard. (A) Lanes 1–8, *Botrytis cinerea*. (B) Lane 1, *Mucor* sp.; lanes 2–4, *Penicillium* spp.; lanes 5–9, *Pythium* spp.; lanes 10–12, *Fusarium* spp.

**Figure S3**. Microbes are prevalent on cannabis inflorescences and in the pith tissues. (A), (B) Petri dish drop plate assay recovers airborne microbes. Plates are exposed for 60 min in the growing environment. (C), (D) The swab method recovers microbes on inflorescence surfaces. Cotton swabs were wiped across the bud surface and transferred to agar medium. (E)–(H) The pith tissues of cannabis stems contain endophytic *Penicillium* spp. (E) Section of stem with central pith surrounded by a ring of parenchyma cells. (F) Stem section under the scanning electron microscope. (G) *Penicillium* spp. emerge from surface‐sterilized stem segments plated on agar medium. (H) *Penicillium* sporulates on central pith cells (scanning electron microscope).

**Figure S4**. Powdery mildew on cannabis plants is managed by reduced risk products. Treatments were applied weekly for 4 weeks: (A) untreated control, (B) *Streptomyces lydicus* strain WYEC 43, (C) hydrogen peroxide, (D) *Bacillus subtilis* strain QST 713, (E) plant extract from giant knotweed, (F) potassium bicarbonate, (G) leaves received daily exposure to UV‐C for 3–5 s over 28 days, (H) untreated control.

**Figure S5**. Biological control agents reduce *Fusarium* development on rooted stem cuttings of cannabis: (A) *Trichoderma harzianum*, (B) *Gliocladium catenulatum*, (C) *Trichoderma asperellum* were applied at recommended rates 48 h prior to pathogen inoculation. (D) *Fusarium* control. Photos were taken 14 days after treatment. (E)–(H) Respective biocontrol fungi and *Fusarium* are recovered from internal stem tissues 14 days after application, suggesting endophytic colonization.

**Figure S6**. Botrytis bud rot is inhibited by biological control agents: (A) Antagonism by *Bacillus amyloliquefaciens*, (B) *Trichoderma asperellum in vitro*. (C), (D). Buds treated with the same biocontrols 48 h prior to inoculation with the pathogen show reduced development of disease after 7 days. The results were consistent over repeated experimental trials.
