# Supplementary material for: Emerging diseases of Cannabis sativa and sustainable management
Source: Pest Manag Sci. 2021 Feb 27;77(9):3857–70. doi: 10.1002/ps.6307 (PMC8451794; doi:10.1002/ps.6307)
Supplement: Supplementary file 1 — Table S1. Emerging pathogens reported on cannabis and hemp plants during 2017–2020. [file PS-77-3857-s002.pdf]

https://doi.org/10.1002/ps.6307

Supplementary Table 1. Emerging pathogens reported on cannabis and hemp plants during 2017-2020.

Common name of disease	Pathogen	Crop plant: Cannabis (Indoor/ Outdoor*)	Crop plant: Hemp (Outdoor)	Reference
Bud rots	*Alternaria alternata*	+*	+	1, 2 , unpublished
	Botrytis cinerea	+	+	1, 2, 3, 4, 5, 6, 7, 8
	Botrytis pseudocinerea	+*	+	9; this study
	Botrytis porri	+*	-	Unpublished
	Chaetomium globosum	+	-	Unpublished
	Diaporthe eres/ D. subordinaria	+	-	Unpublished
	Fusarium equiseti	+	+	1, 2, 10, 11
	Fusarium graminearum	+	+	2, 3, 11, unpublished
	Fusarium oxysporum	+	-	6, 7, 10, 12
	Fusarium proliferatum	+	-	6, 13
	Fusarium solani	+	-	10
	Fusarium sporotrichiodes	+	-	6
	Phoma multirostrata	+	-	Unpublished
	Sclerotinia sclerotiorum	+	-	Unpublished
Crown and root rots and wilts	*Fusarium avenaceum*	+	+	6, unpublished
	Fusarium brachygibbosum	+*	-	8, 14
	Fusarium graminearum	-	+	3, 15
	Fusarium lichenicola	+	-	16
	Fusarium oxysporum	+	+	1, 2, 8, 11, 12, 15, 17, 18
	Fusarium proliferatum	+	-	1,13
	Fusarium solani	+	+	1, 17, 19
	Fusarium tricinctum	+	+	6, unpublished
	Globisporangium irregulare	+	+	18, unpublished
	Globisporangium ultimum	+	+	2, 11, 20, 21
	Phytophthora sp.	+	-	Unpublished
	Pythium aphanidermatum	+	+	11, 17, 19, 21, 22
	Pythium catenulatum	+	-	8, 21
	Pythium dissotocum	+	-	19, 21
	Pythium myriotylum	+	+	19, 21, 23

https://doi.org/10.1002/ps.6307

	Rhizoctonia solani	+	+	2, 3, 11, 15, 24
	Sclerotinia minor	-	+	25
	Thielaviopsis basicola	-	+	Unpublished
	Verticillium dahliae/V.albo-atrum	+	+	26, unpublished
**Crown gall**	*Agrobacterium tumefaciens*	+	-	Unpublished
**Damping-off**	*Alternaria alternata*	-	+	2, unpublished
	Botrytis cinerea	+	+	2, 12, 10, 11, 17
	Fusarium oxysporum	+	+	1, 2, 8, 12, 17
	Fusarium proliferatum	+	-	13, 27
	Fusarium solani	+	+	16
	Fusarium sporotrichiodes	+	-	6
	Pythium aphanidermatum	+	+	8, 20
	Sclerotinia sclerotiorum	+	+	2, 3, 15
	Stemphylium vesicarium	-	+	Unpublished
**Downy mildew**	*Pseudoperonospora humuli*	-	+	28
**Fungal leaf spots, foliar blight**	*Alternaria alternata*	-	+	1, 2
	Bipolaris gigantea	-	+	2, 3, 17, 29
	Botrytis cinerea	+	-	1, 2, unpublished
	Cercospora cf. flagellaris	-	+	30, 31, 32
	Chaetomium globosum	-	+	33
	Colletotrichum fioriniae	-	+	34
	Curvularia pseudobrachyspora	-	+	17, 31
	Rhizoctonia solani	-	+	24
	Septoria sp.	-	+	2, 3
**Bacterial leaf spots**	*Pseudomonas koreensis*	-	+	11
	Serratia marcescens	-	+	11, 35
	Sphingomonas yanoikuyae	-	+	11
**Post-harvest rots**	*Botrytis cinerea*	+	+	1, 6, 8, 10, 36
	Chaetomium globosum	+*	-	Unpublished
	Penicillium spp.	+	-	1, 6, 8, 10
	Fusarium oxysporum	+	-	6, 10, 12
	Fusarium proliferatum	+	-	6, 13
	Fusarium sporotrichiodes	+	-	6
**Powdery mildew**	*Golovinomyces cichoracearum*	+	+	8, 10, 17, 37
	Golovinomyces ambrosiae	-	+	37

	Golovinomyces spadiceus	-	+	38, 39
	Podosphaeria macularis	+	+	9, 40, 41, 42
**Stem canker / dieback**	*Alternaria alternata*	+	+	Unpublished
	Botrytis cinerea	+	+	4, 8
	Diaporthe eres	+	-	Unpublished
	Fusarium chlamydosporum	-	+	17
	Fusarium graminearum	-	+	2, 11, unpublished
	Lasiodiplodia theobromae	+	+	17, unpublished
	Neofusicoccum parvum	+*	+	17, unpublished
	Phoma multirostrata	+	-	Unpublished
	Sclerotinia sclerotiorum	+	+	2, 3, 31, 43
**Southern blight**	*Sclerotium rolfsii*	-	+	2, 11, 15, 44, 45
**Viruses/viroids**	Beet curly top virus	-	+	46, 47
	Cannabis cryptic virus	+	+	47, 48
	Cannabis sativa mitovirus 1	-	+	47
	Citrus yellow-vein associated virus	-	+	47
	Hop latent viroid	+	+	47, 49, 50
	Lettuce chlorosis virus	+	+	51
	Tobacco streak virus	-	+	46
**Nematodes**	Root knot nematodes (*Meloidogyne incognita*)	-	+	2, 11

Unpublished = author's personal observations confirmed by pathogenicity tests.

+ = pathogen detected; - = pathogen absent based on published reports. All identifications reported are based on molecular methods that include PCR and sequencing, with pathogenicity testing where available.

* Refers to outdoor grown cannabis.

REFERENCES

1 Jerushalmi S, Maymon M, Dombrovsky and Freeman S, Fungal pathogens affecting the production and quality of medical cannabis in Israel. *Plants* **9**, 882 (2020). doi:10.3390/plants9070882.

2 Science of Hemp: Production and Pest Management, Proceedings of the first annual scientific conference, by eds. Gauthier N, Leonberger K, Bowers K, Publication SR-112, College of Agriculture, Food and Environment, University of Kentucky, Lexington (2020). https://plantpathology.ca.uky.edu/files/sr112.pdf [accessed on July 12 2020].

https://doi.org/10.1002/ps.6307

3 Bergstrom G, Starr J and Myers K, Diseases affecting hemp in New York. Cornell College of Agriculture and Life Sciences (2020). https://cpb-us-e1.wpmucdn.com/blogs.cornell.edu/dist/a/7491/files/2020/09/2020-HempDiseaseHandout_compressed.pdf [accessed 8 October 2020].

4 Garfinkel AR, Three *Botrytis* species found causing gray mold on industrial hemp (*Cannabis sativa)* in Oregon. *Plant Dis* **104**:2026 (2020).

5 Jerushalmi S, Maymon M, Dombrovsky A and Freeman S, Effects of cold plasma, gamma and e-beam irradiations on reduction of fungal colony forming unit levels in medical cannabis inflorescences. *J Cannabis Res* **2**:12 (2020).

6 Punja ZK, The diverse mycoflora present on dried cannabis (*Cannabis sativa* L.) inflorescences in commercial production. *Can J Plant Pathol* (2021). doi: 10.1080/07060661.2020.1758959.

7 Punja ZK, Diseases that can devastate *Cannabis sativa* L. – root and crown rots, powdery mildew and bud rots. *Can J Plant Path* (2020) **42** (abstr.) (2020).

8 Punja ZK, Collyer D, Scott C, Lung S, Holmes J and Sutton D, Pathogens and molds affecting production and quality of *Cannabis sativa* L. *Front Plant Sci* (2019) https://doi.org/10.3389/fpls.2019.01120.

9 Garfinkel A, Multiple *Botrytis* and powdery mildew species associated with industrial hemp in Oregon. Amer Phytopath Soc Ann Meet abstr. (2020). https://apsnet.confex.com/apsnet/2020/meetingapp.cgi/Paper/16975 [accessed 8 August 2020].

10 Punja ZK, Flower and foliage-infecting pathogens of marijuana (*Cannabis sativa* L.) plants. *Can J Plant Pathol* **40**:514-527 (2018).

11 Thiessen LD, Schappe T, Cochran S, Hicks K and Post AR, Surveying for potential diseases and abiotic disorders of industrial hemp (*Cannabis sativa* L.) production. *Plant Health Progress* **21**:321-332 (2020). doi/10.1094/PHP-03-20-0017-RS.

12 Punja ZK, Epidemiology of *Fusarium oxysporum* causing root and crown rot of cannabis (*Cannabis sativa* L., marijuana) plants in commercial greenhouse production. *Can J. Plant Pathol* (2021).doi: 10.1080/07060661.2020.1788165.

13 Punja ZK, First report of *Fusarium proliferatum* causing crown and stem rot, and pith necrosis, in cannabis *(Cannabis sativa*, L., marijuana) plants. *Can J Plant Pathol* (2021). doi: 10.1080/07060661.2020.1793222.

14 Punja ZK, Scott C and Chen S, Root and crown rot pathogens causing wilt symptoms on field-grown marijuana (*Cannabis sativa* L.) plants. *Can J Plant Pathol* **40**: 528-541 (2018).

15 Thiessen L, Root diseases prevalent in industrial hemp (2019). https://plantpathology.ces.ncsu.edu/2019/08/root-diseases-prevalent-in-industrial-hemp/ [accessed on 25 September 2020].

16 Punja ZK, Brown root rot and crown rot of cannabis (*Cannabis sativa* L., marijuana) plants caused by *Fusarium* (*Cylindrocarpon*) *lichenicola*. Amer Phytopath Soc Ann Meet abstr. (2020). https://apsnet.confex.com/apsnet/2020/meetingapp.cgi/Paper/16064 [accessed 3 September 2020].

17 Feng C, Villarroel-Zeballos M, Ficheaux P, Zima H and Correll J, Hemp diseases in Arkansas. Amer Phytopath Soc Ann Meet abstr. (2020). https://apsnet.confex.com/apsnet/2020/meetingapp.cgi/Paper/16685 [accessed 7 August 2020].

18 McGehee CS and Raudales RE, Characterization of oomycetes and fungi from the substrate of marijuana *(Cannabis sativa* L.) plants. Amer Phytopath Soc Ann Meet abstr. (2020). https://apsnet.confex.com/apsnet/2020/meetingapp.cgi/Paper/17488 [accessed 2 October 2020].

19 Punja ZK and Rodriguez G, *Fusarium* and *Pythium* species infecting roots of hydroponically grown marijuana (*Cannabis sativa* L.) plants. *Can J Plant Pathol* **40**:498-513 (2018).

20 Beckerman J, Stone J, Ruhl G and Creswell T, 2018. First report of *Pythium ultimum* crown and root rot of industrial hemp in the United States. *Plant Dis* **102**: 2045 (2018).

21 Punja ZK, Scott C, Lung S and Roberts A, *Pythium* species associated with crown and root rot on cannabis (*Cannabis sativa* L., marijuana) plants grown under commercial greenhouse conditions. Amer Phytopath Soc Ann Meet abstr. (2020). https://apsnet.confex.com/apsnet/2020/meetingapp.cgi/Paper/16853 [accessed 16 September 2020].

22 Beckerman J, Nisonson H, Albright N and Creswell T, First report of *Pythium aphanidermatum* crown and root rot of industrial hemp in the United States. *Plant Dis* **101**:1038 (2017).

23 McGehee CS, Apicella P, Raudales R, Berkowitz G, Ma Y, Durocher S and Lubell J, First report of root rot and wilt caused by *Pythium myriotylum* on hemp (*Cannabis sativa* L.) in the United States. *Plant Dis* **103**: 3288 (2019).

24 Pacific Northwest Plant Disease Management Handbook. Hemp (*Cannabis sativa)* – Rhizoctonia soreshin and root rot, by eds. Pscheidt JW and Ocamb CM, Oregon State University, Corvallis, OR (2020). https://pnwhandbooks.org/plantdisease/host-and-disease-descriptions?title=Cannabis+sativa [accessed 30 August 2020].

25 Koike ST, Stanghellini H, Mauzey SJ and Burkhardt A, First report of sclerotinia crown rot caused by *Sclerotinia minor* on hemp. *Plant Dis* **103**:1771 (2019).

26 Pacific Northwest Pest Management Handbook. Hemp (*Cannabis sativa)* – Verticillium wilt, by eds. Pscheidt JW and Ocamb CM, Oregon State University, Corvallis, OR (2020). https://pnwhandbooks.org/plantdisease/host-and-disease-descriptions?title=Cannabis+sativa [accessed 30 August 2020].

27 Lung S, Betz EC, Roberts AJ and Punja ZK, Infection of *Cannabis sativa* cuttings by *Fusarium oxysporum* and *Fusarium proliferatum* and investigation into potential biofungicide control. *Can J Plant Pathol* **42**: 461 (abstr.) (2020).

28 Plant Disease Diagnostic Clinic, Cornell University. Mildew of hops: *Podosphaera* and *Pseudoperonospora* (2018), Ithaca, NY. http://plantclinic.cornell.edu/factsheets/mildewsofhops.pdf [accessed 30 August 2020].

29 Szarka D, Amsden B, Beale J, Dixon E, Schardl CL and Gauthier N, First report of hemp leaf spot caused by a *Bipolaris* species on hemp (*Cannabis sativa*) in Kentucky. *Plant Health Progress* **21**:82-84 (2020).

30 Doyle VP, Tonry HT, Amsden B, Beale J, Dixon E, Li H, Szarka D and Gauthier NW, First report of *Cercospora* cf. *flagellaris* on industrial hemp (*Cannabis sativa*) in Kentucky. *Plant Dis* **103**:1784 (2019).

31 Marin M, Wang N-Y, Coburn J, Desaeger J and Peres N, Etiology of emerging leaf spot diseases on industrial hemp (*Cannabis sativa*) in Florida. Amer Phytopath Soc Ann Meet abstr. (2020). https://apsnet.confex.com/apsnet/2020/meetingapp.cgi/Paper/16959 [accessed 8 July 2020].

32 Martin MV, Coburn J, Desaeger J and Peres NA, First report of Cercospora leaf spot caused by *Cercospora* cf. *flagellaris* on industrial hemp in Florida. *Plant Dis* **104**:1536 (2020).

33 Chaffin AG, Dee ME, Boggess SL, Trigiano RN, Bernard EC and Gwinn KD, First report of *Chaetomium globosum* causing a leaf spot of hemp (*Cannabis sativa)* in Tennessee. *Plant Dis* **104** (2020). https://doi.org/10.1094/PDIS-08-19-1697-PDN.

34 Szarka D, McCulloch M, Beale J, Long S, Dixon E and Gauthier N, First report of anthracnose leaf spot caused by *Colletotrichum fioriniae* on hemp (*Cannabis sativa)*. *Plant Dis* **104**:1560 (2020).

35 Schappe TL, Ritchie D and Thiessen LD, First report of *Serratia marcescens* casing a leaf spot on industrial hemp (*Cannabis sativa*). *Plant Dis* **104**:1248.

36 Punja ZK, Cannabis and hemp biology and pathology: an overview of the crop and emerging pathogens. Amer Phytopath Soc Ann Meet abstr. (2020). https://apsnet.confex.com/apsnet/2020/meetingapp.cgi/Paper/15576 [accessed 3 September 2020].

37 Pépin N, Punja ZK and Joly DL, Occurrence of powdery mildew caused by *Golovinomyces cichoracearum sensu lato* on *Cannabis sativa* in Canada. *Plant Dis* **102**:2644-2644 (2018).

38 Cala AR, Day CTC, Giles G, Carlson C, Stack G, Ullrich M, Crawford J, Smart L and Smart CD, Evaluation of hemp powdery mildew host resistance and host range. Amer Phytopath Soc Ann Meet abstr. (2020). https://apsnet.confex.com/apsnet/2020/meetingapp.cgi/Paper/16386 [accessed 30 September 2020].

39 Szarka D, Tymon L, Amsden B and Dixon E, First report of powdery mildew caused *by Golovinomyces spadiceus* on industrial hemp (*Cannabis sativa)* in Kentucky. *Plant Dis* **103**: 1773 (2019).

https://doi.org/10.1002/ps.6307

40 Gent DH, Nelson ME, George AE, Grove GG, Mahaffee WF, Ocamb CM, et al., A decade of hop powdery mildew in the Pacific northwest. *Plant Health Progress* **9** (2018).

41 Punja ZK, First report of the hops powdery mildew pathogen, *Podosphaeria macularis*, on naturally infected marijuana (*Cannabis sativa* L.) plants in the field. Amer Phytopath Soc Ann Meet abstr. (2020). https://apsnet.confex.com/apsnet/2020/meetingapp.cgi/Paper/16065 [accessed 7 September 2020].

42 Weldon WA, Ullrich MR, Smart LB, Smart CD and Gadoury DM, Cross-infectivity of powdery mildew isolates originating from hemp (*Cannabis sativa)* and Japanese hop *(Humulus japonicus)* in New York. *Plant Health Progress* **21**:47-53 (2020).

43 Bain PS, Bennypaul HS, Blade SF and Weeks C, First report of hemp canker caused by *Sclerotinia sclerotiorum* in Alberta, Canada. *Plant Dis* **84**:372 (2007).

44 Mersha Z, Kering M and Ren S, Southern blight of hemp caused by *Athelia rolfsii* detected in Virginia. *Plant Dis* **104**:1562 (2020).

45 Morgan J, Louisiana: industrial hemp hit hard by southern blight (2020). https://agfax.com/2020/07/24/louisiana-industrial-hemp-hit-hard-by-southern-blight/ [accessed 17 August 2020].

46 Giladi Y, Hadad L, Luria N, Cranshaw W, Lachman O and Dombrovsky A, First report of beet curly top virus infecting *Cannabis sativa* in western Colorado. *Plant Dis* **104** (2020). https://doi.org/10.1094/PDIS-08-19-1656-PDN.

47 Nachappa P, Fulladolsa AC and Stenglein M, Wild wild west: emerging viruses and viroids of hemp. *Outlooks Pest Manag* **31**:175-179 (2020).

48 Righetti L, Paris R, Ratti C, Calassanzio M, Onofri C, Calzolari D, et al., Not the one, but the only one: about *Cannabis cryptic virus* in plants showing ‘hemp streak’ disease symptoms. *Eur J Plant Pathol* **150**: 575-588 (2017).

49 Bektas A, Hardwick KM, Waterman K and Kristof J, Occurrence of hop latent viroid in *Cannabis sativa* with symptoms of cannabis stunting disease in California. *Plant Dis* **103**: 2699 (2019).

50 Warren JG, Mercado J and Grace D, Occurrence of hop latent viroid causing disease in *Cannabis sativa* in California. *Plant Dis* **103:** 2699 (2019).

51 Hadad L, Luria N, Smith E, Sela N, Lachman O and Dombrovsky A, Lettuce chlorosis virus disease: a new threat to cannabis production. *Viruses* **11**:802 (2019).
