# Supplementary figures and images for: Emerging diseases of Cannabis sativa and sustainable management
Source: Pest Manag Sci. 2021 Feb 27;77(9):3857–70. doi: 10.1002/ps.6307 (PMC8451794; doi:10.1002/ps.6307)

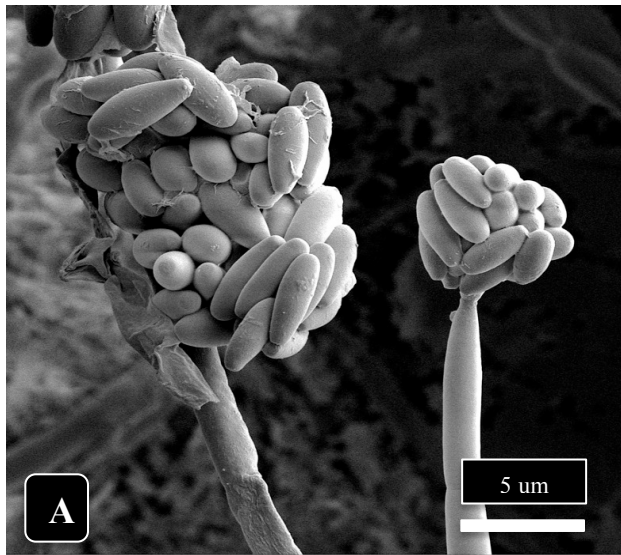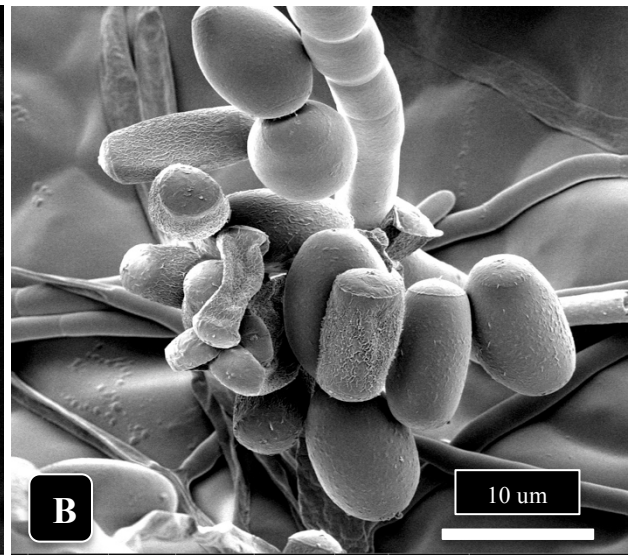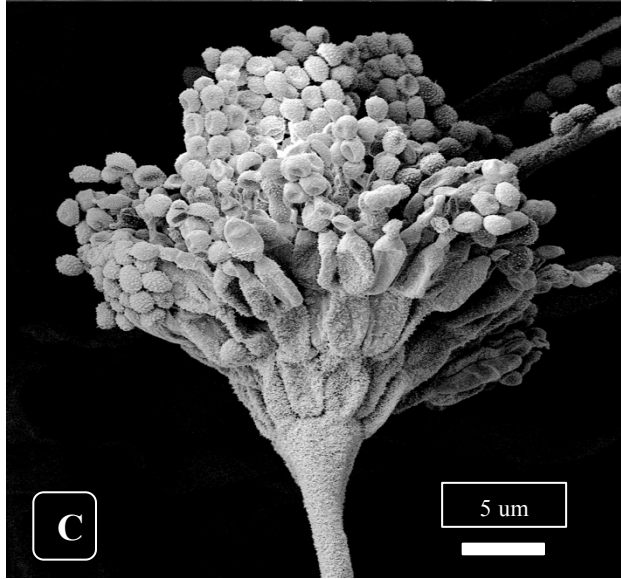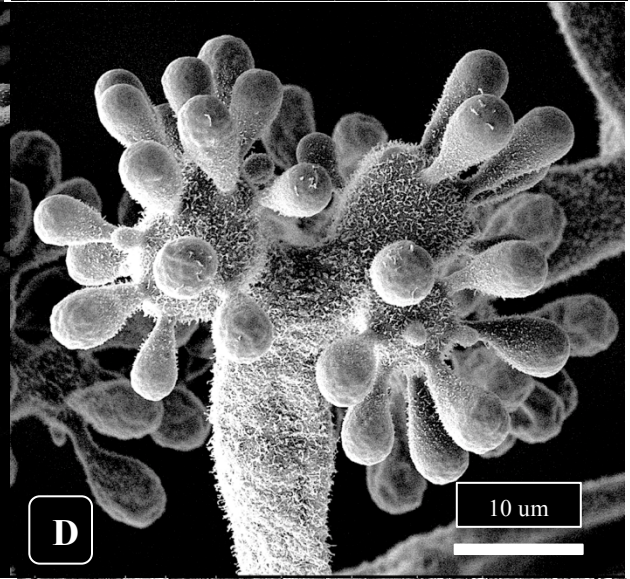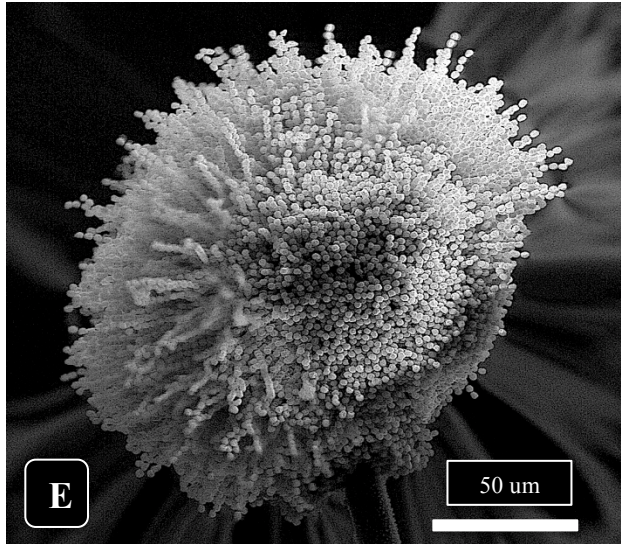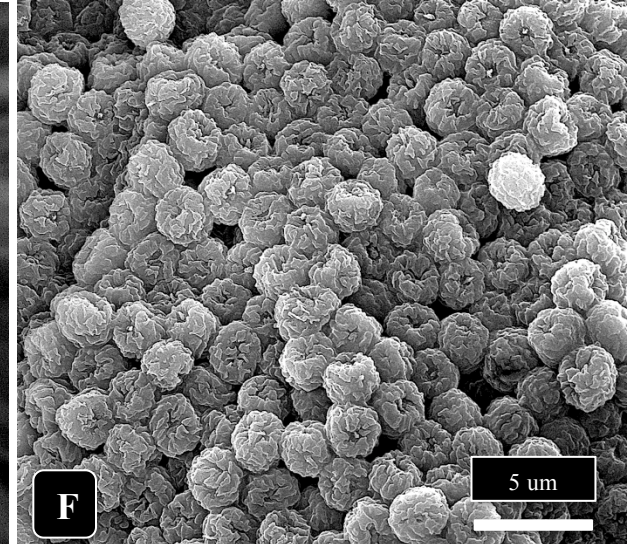

Supplement: Supplementary file 2 — Figure S1. Spore production by the most important pathogens affecting cannabis and hemp production: (A) Fusarium oxysporum, (B) Botrytis cinerea, (C) Golovinomyces species, (D) Penicillium species, (E, F) Aspergillus sp. (A)–(D) are reproduced from the Canadian Journal of Plant Pathology by permission from the Canadian Phytopathological Society. [file PS-77-3857-s004.pdf]

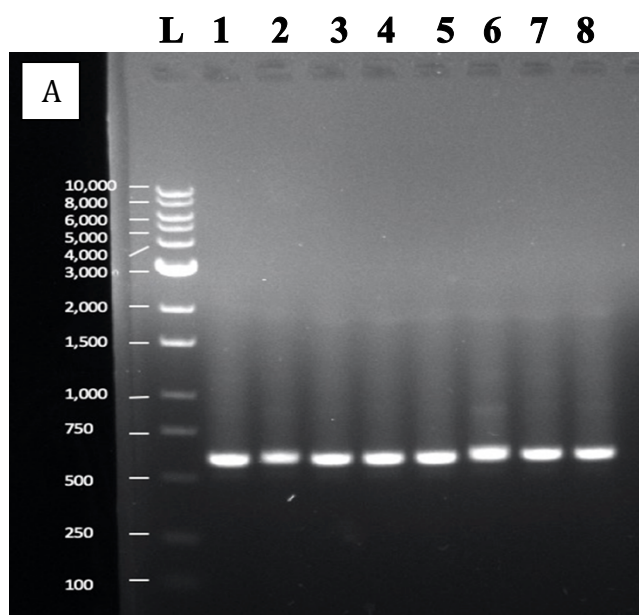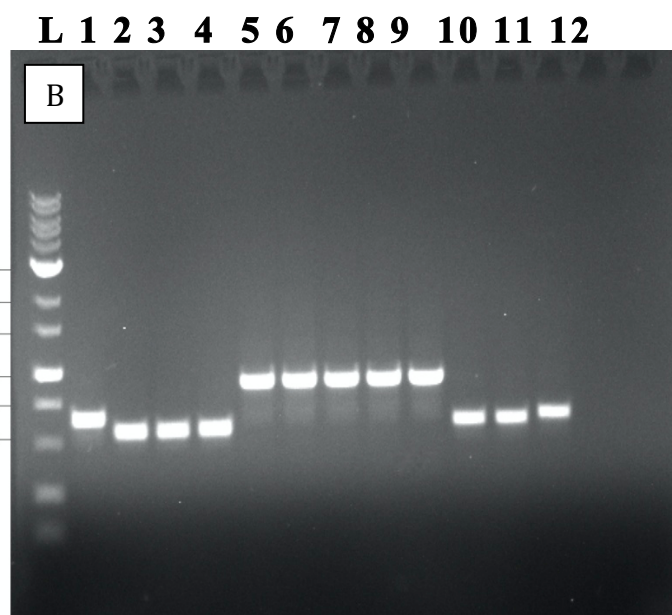

Supplement: Supplementary file 3 — Figure S2. Gel electrophoresis of PCR products after amplification of DNA with primers for the internal transcribed spacer region (ITS1‐5.8S‐ITS2) of rDNA. Lane L, molecular weight standard. (A) Lanes 1–8, Botrytis cinerea. (B) Lane 1, Mucor sp.; lanes 2–4, Penicillium spp.; lanes 5–9, Pythium spp.; lanes 10–12, Fusarium spp. [file PS-77-3857-s003.pdf]

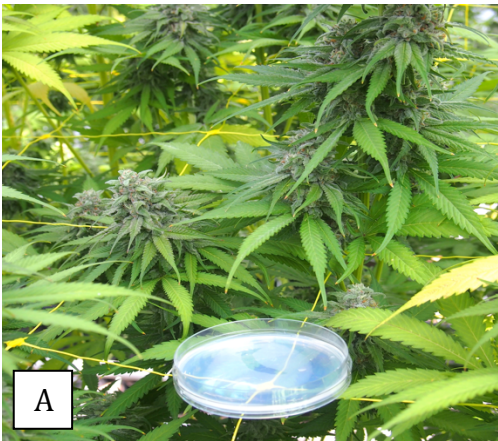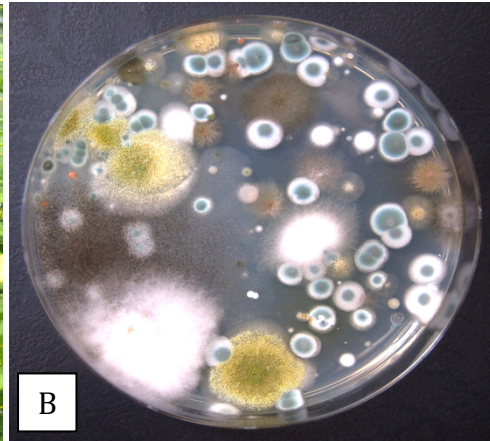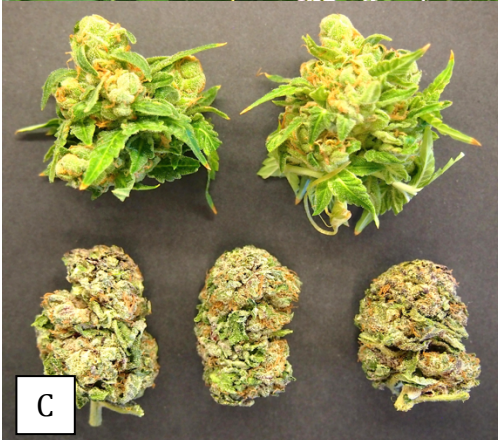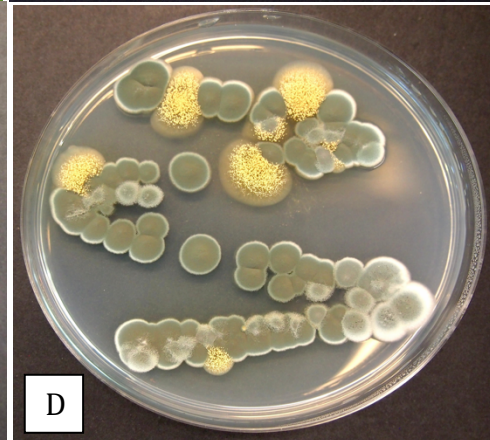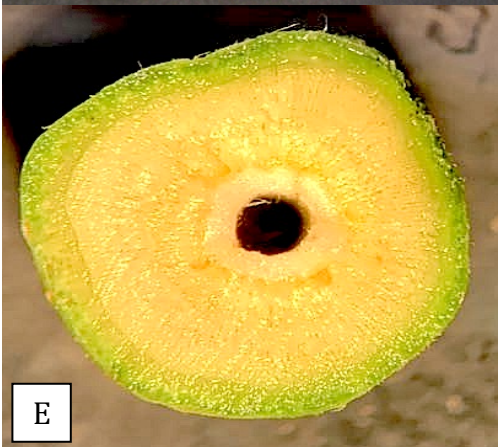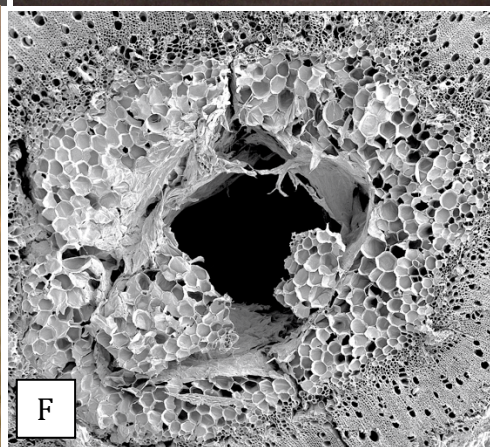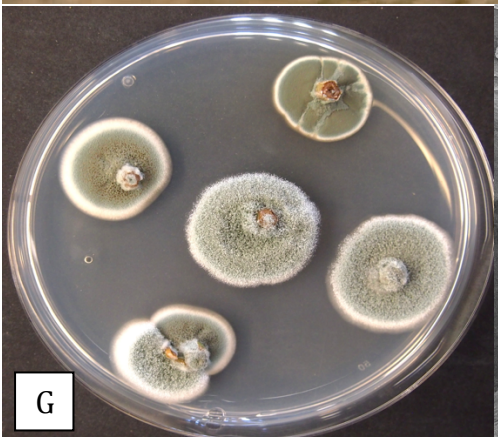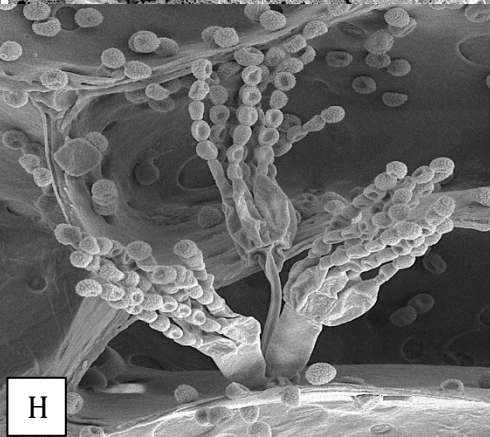

Supplement: Supplementary file 4 — Figure S3. Microbes are prevalent on cannabis inflorescences and in the pith tissues. (A), (B) Petri dish drop plate assay recovers airborne microbes. Plates are exposed for 60 min in the growing environment. (C), (D) The swab method recovers microbes on inflorescence surfaces. Cotton swabs were wiped across the bud surface and transferred to agar medium. (E)–(H) The pith tissues of cannabis stems contain endophytic Penicillium spp. (E) Section of stem with central pith surrounded by a ring of parenchyma cells. (F) Stem section under the scanning electron microscope. (G) Penicillium spp. emerge from surface‐sterilized stem segments plated on agar medium. (H) Penicillium sporulates on central pith cells (scanning electron microscope). [file PS-77-3857-s001.pdf]

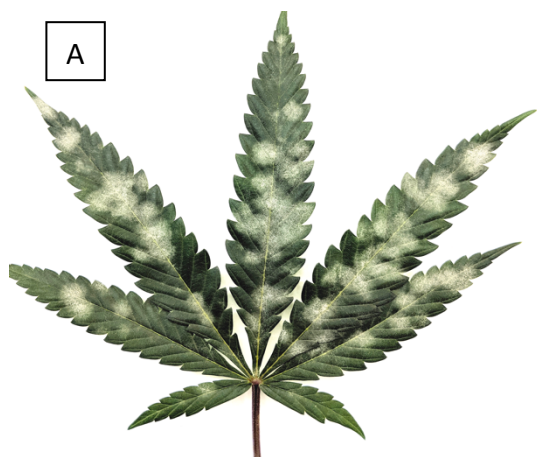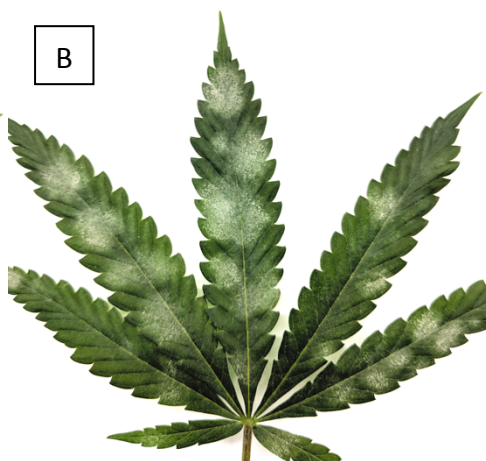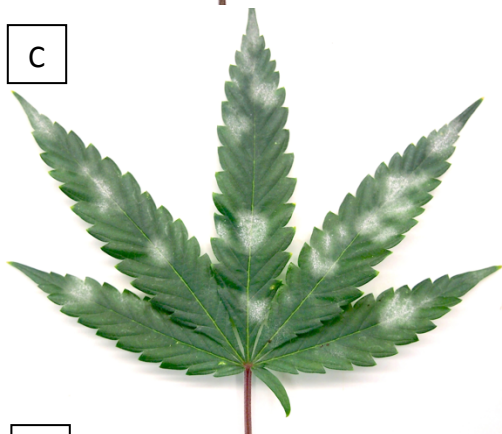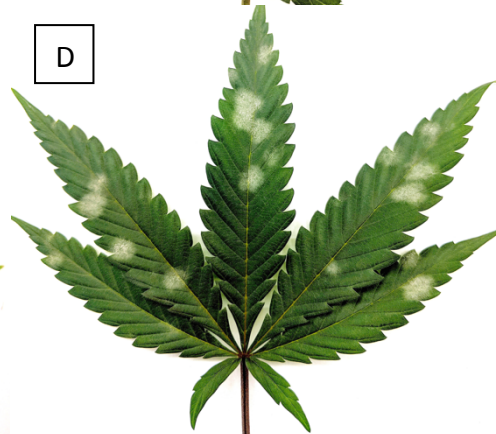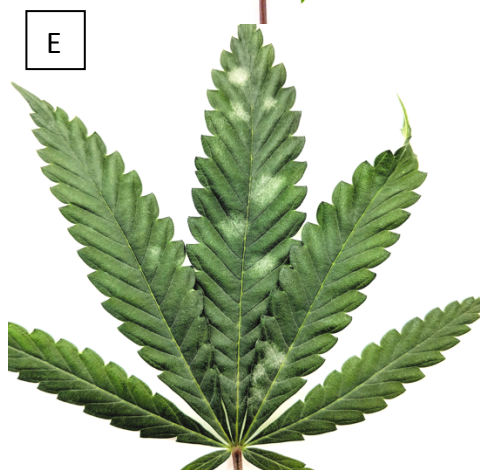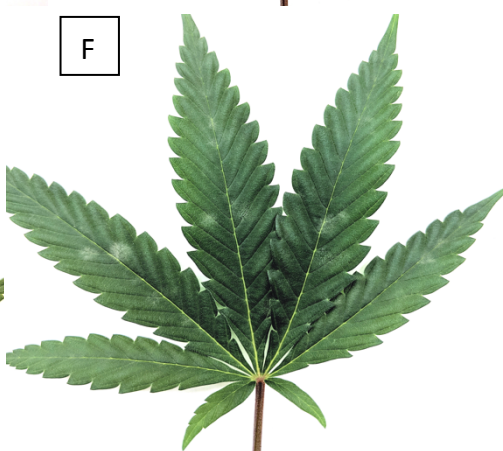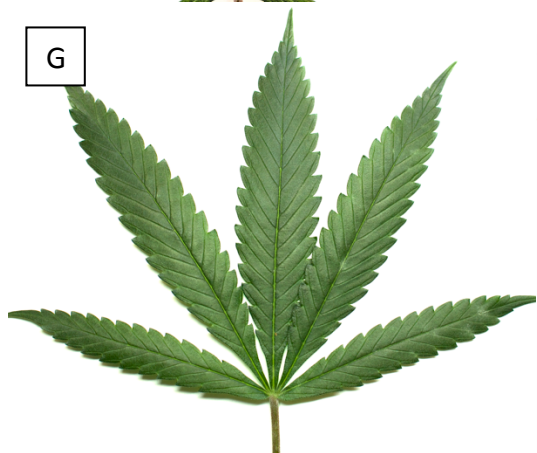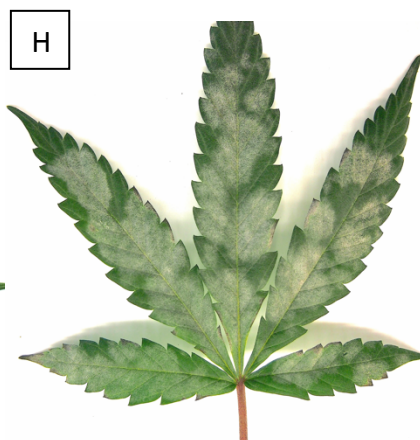

Supplement: Supplementary file 5 — Figure S4. Powdery mildew on cannabis plants is managed by reduced risk products. Treatments were applied weekly for 4 weeks: (A) untreated control, (B) Streptomyces lydicus strain WYEC 43, (C) hydrogen peroxide, (D) Bacillus subtilis strain QST 713, (E) plant extract from giant knotweed, (F) potassium bicarbonate, (G) leaves received daily exposure to UV‐C for 3–5 s over 28 days, (H) untreated control. [file PS-77-3857-s007.pdf]

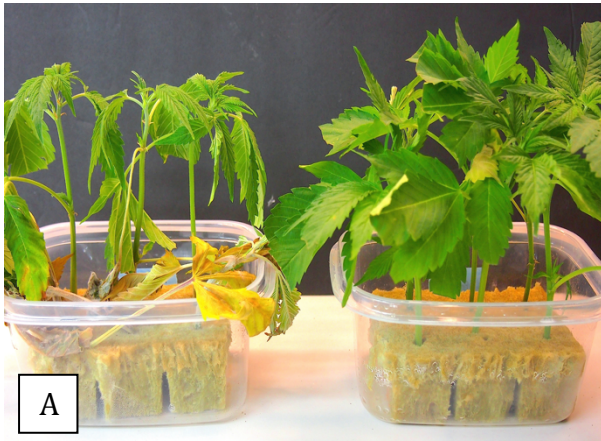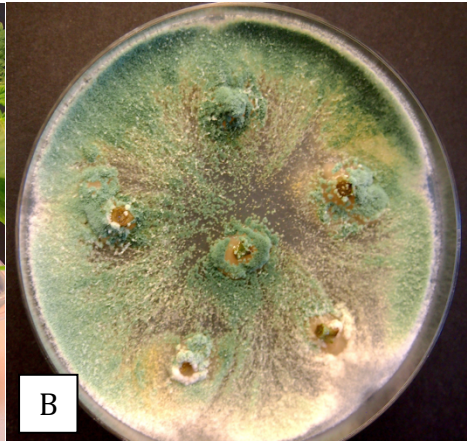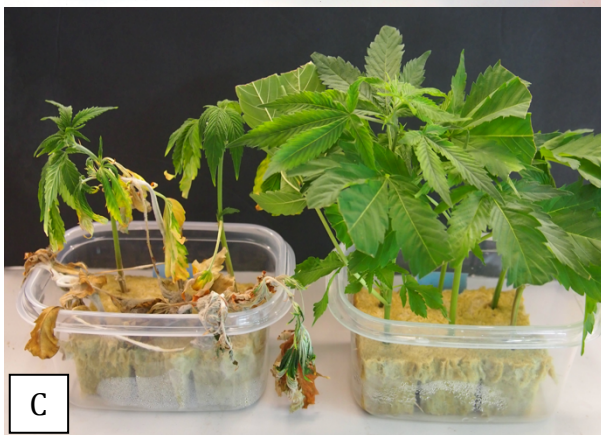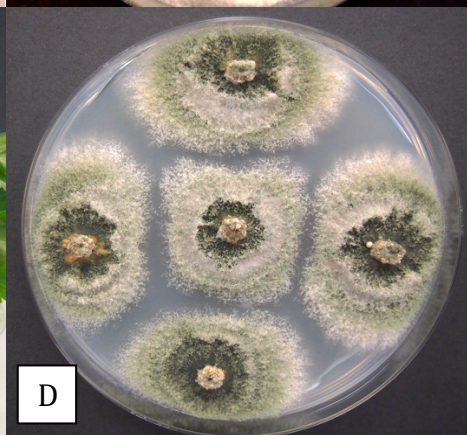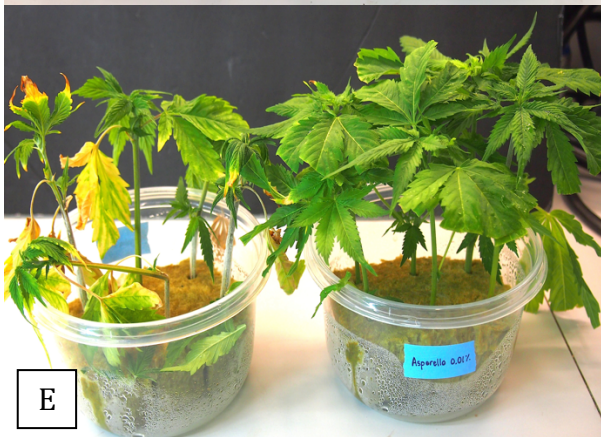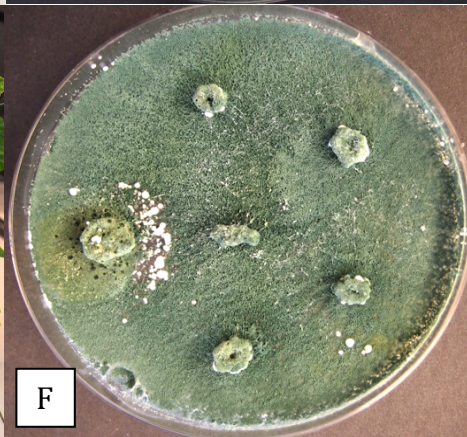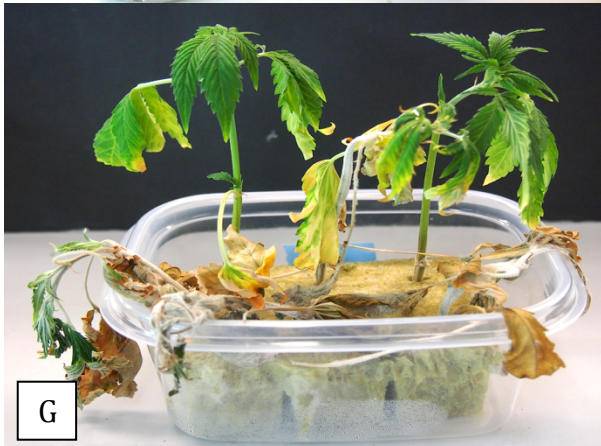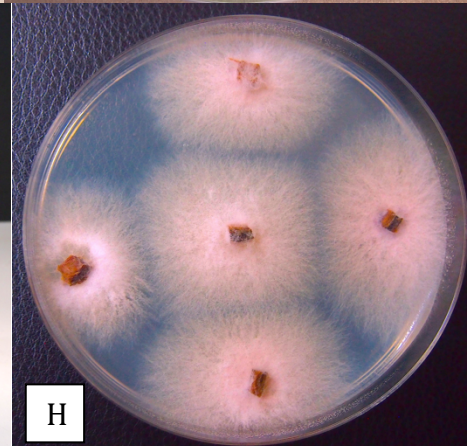

Supplement: Supplementary file 6 — Figure S5. Biological control agents reduce Fusarium development on rooted stem cuttings of cannabis: (A) Trichoderma harzianum, (B) Gliocladium catenulatum, (C) Trichoderma asperellum were applied at recommended rates 48 h prior to pathogen inoculation. (D) Fusarium control. Photos were taken 14 days after treatment. (E)–(H) Respective biocontrol fungi and Fusarium are recovered from internal stem tissues 14 days after application, suggesting endophytic colonization. [file PS-77-3857-s005.pdf]

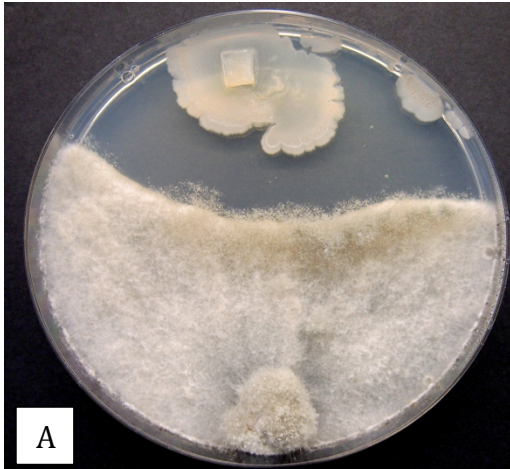

A

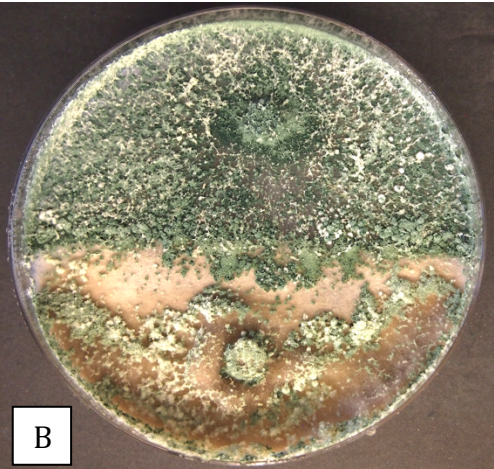

B

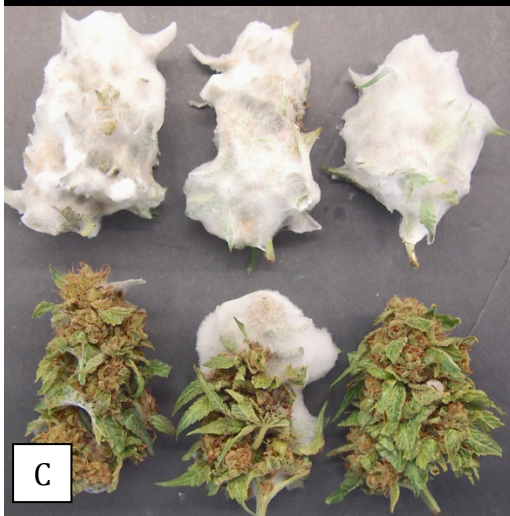

C

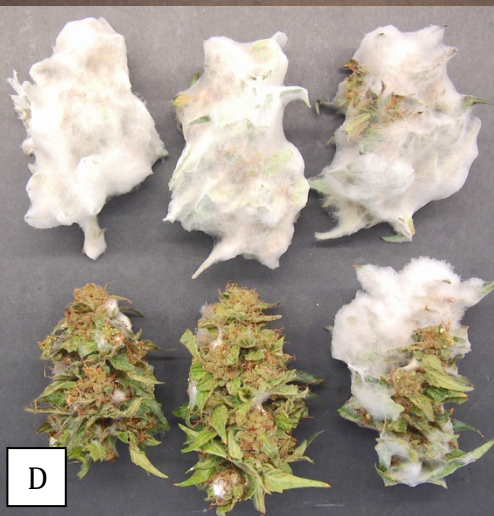

D

Supplement: Supplementary file 7 — Figure S6. Botrytis bud rot is inhibited by biological control agents: (A) Antagonism by Bacillus amyloliquefaciens, (B) Trichoderma asperellum in vitro. (C), (D). Buds treated with the same biocontrols 48 h prior to inoculation with the pathogen show reduced development of disease after 7 days. The results were consistent over repeated experimental trials. [file PS-77-3857-s006.pdf]
